# CARM1 drives triple-negative breast cancer progression by coordinating with HIF1A

**DOI:** 10.1093/procel/pwae010

**Published:** 2024-03-13

**Authors:** Dandan Feng, Jie Gao, Ruiqiong Liu, Wei Liu, Tianyang Gao, Yunkai Yang, Die Zhang, Tianshu Yang, Xin Yin, Hefen Yu, Wei Huang, Yan Wang

**Affiliations:** Key Laboratory of Cancer and Microbiome, State Key Laboratory of Molecular Oncology, National Cancer Center, National Clinical Research Center for Cancer, Cancer Hospital, Chinese Academy of Medical Sciences and Peking Union Medical College, Beijing 100021, China; Key Laboratory of Immune Microenvironment and Disease (Ministry of Education), Department of Biochemistry and Molecular Biology, School of Basic Medical Sciences, Tianjin Medical University, Tianjin 300070, China; Department of Clinical Laboratory, The Second Hospital of Shandong University, Jinan 250033, China; Department of Clinical Laboratory, The Second Hospital of Shandong University, Jinan 250033, China; Department of Cancer Center, The Second Hospital of Shandong University, Jinan 250033, China; Key Laboratory of Immune Microenvironment and Disease (Ministry of Education), Department of Biochemistry and Molecular Biology, School of Basic Medical Sciences, Tianjin Medical University, Tianjin 300070, China; Key Laboratory of Immune Microenvironment and Disease (Ministry of Education), Department of Biochemistry and Molecular Biology, School of Basic Medical Sciences, Tianjin Medical University, Tianjin 300070, China; Key Laboratory of Cancer and Microbiome, State Key Laboratory of Molecular Oncology, National Cancer Center, National Clinical Research Center for Cancer, Cancer Hospital, Chinese Academy of Medical Sciences and Peking Union Medical College, Beijing 100021, China; Key Laboratory of Cancer and Microbiome, State Key Laboratory of Molecular Oncology, National Cancer Center, National Clinical Research Center for Cancer, Cancer Hospital, Chinese Academy of Medical Sciences and Peking Union Medical College, Beijing 100021, China; Beijing Key Laboratory of Cancer Invasion and Metastasis Research, Department of Biochemistry and Molecular Biology, School of Basic Medical Sciences, Capital Medical University, Beijing 100069, China; Beijing Key Laboratory of Cancer Invasion and Metastasis Research, Department of Biochemistry and Molecular Biology, School of Basic Medical Sciences, Capital Medical University, Beijing 100069, China; Beijing Key Laboratory of Cancer Invasion and Metastasis Research, Department of Biochemistry and Molecular Biology, School of Basic Medical Sciences, Capital Medical University, Beijing 100069, China; Beijing Key Laboratory of Cancer Invasion and Metastasis Research, Department of Biochemistry and Molecular Biology, School of Basic Medical Sciences, Capital Medical University, Beijing 100069, China; Key Laboratory of Cancer and Microbiome, State Key Laboratory of Molecular Oncology, National Cancer Center, National Clinical Research Center for Cancer, Cancer Hospital, Chinese Academy of Medical Sciences and Peking Union Medical College, Beijing 100021, China; Key Laboratory of Immune Microenvironment and Disease (Ministry of Education), Department of Biochemistry and Molecular Biology, School of Basic Medical Sciences, Tianjin Medical University, Tianjin 300070, China; Beijing Key Laboratory of Cancer Invasion and Metastasis Research, Department of Biochemistry and Molecular Biology, School of Basic Medical Sciences, Capital Medical University, Beijing 100069, China

**Keywords:** CARM1, HIF1A, CDK4, ellagic acid, TNBC

## Abstract

Coactivator-associated arginine methyltransferase 1 (CARM1) promotes the development and metastasis of estrogen receptor alpha (ER**α**)-positive breast cancer. The function of CARM1 in triple-negative breast cancer (TNBC) is still unclear and requires further exploration. Here, we report that CARM1 promotes proliferation, epithelial–mesenchymal transition, and stemness in TNBC. CARM1 is upregulated in multiple cancers and its expression correlates with breast cancer progression. Genome-wide analysis of CARM1 showed that CARM1 is recruited by hypoxia-inducible factor-1 subunit alpha (HIF1A) and occupy the promoters of *CDK4*, *Cyclin D1*, ***β**-Catenin*, *HIF1A*, *MALAT1*, and *SIX1* critically involved in cell cycle, HIF-1 signaling pathway, Wnt signaling pathway, VEGF signaling pathway, thereby modulating the proliferation and invasion of TNBC cells. We demonstrated that CARM1 is physically associated with and directly interacts with HIF1A. Moreover, we found that ellagic acid, an inhibitor of CARM1, can suppress the proliferation and invasion of TNBC by directly inhibiting CDK4 expression. Our research has determined the molecular basis of CARM1 carcinogenesis in TNBC and its effective natural inhibitor, which may provide new ideas and drugs for cancer therapy.

## Introduction

For women, breast cancer alone accounting for 31% of female cancers. Female breast cancer incidence rates have been slowly increasing (Siegel et al., 2023). Among different subtypes of breast cancer, triple-negative breast cancer (TNBC) with negative expression of estrogen receptor (ER), progesterone receptor (PR), and human epidermal growth factor receptor 2 (HER2) is more malignant, easy to disseminate, and has no effective treatment and poor prognosis (Garrido-Castro et al., 2019; Kalimutho et al., 2015).

Arginine methylation is reported as a key post-translational modification (PTM) and is involved in various cellular processes, such as mRNA splicing, DNA damage signaling, transcription, and cell signaling (Blanc and Richard, 2017; Jarrold and Davies, 2019). Protein arginine methyltransferases (PRMTs) can catalyze the formation of methylated arginine and S-adenosylhomocysteine. Thus far, three types of modified arginine have been identified: monomethylated arginine (MMA), asymmetric dimethylarginine (ADMA), and symmetric dimethylarginine (SDMA). PRMTs can be divided into three categories according to their catalytic activity: type I PRMTs (PRMT1, 2, 3, 4, 6, and 8), type II (PRMT5), and type III (PRMT7). Type I PRMTs catalyze the formation of MMA and ADMA; type II, MMA and SDMA; and type III, MMA (Fedoriw et al., 2019). The abnormal expression and activity of these PRMTs are associated with tumorigenesis. Therefore, PRMTs have received considerable attention in recent years.

Particularly, the coactivator-associated methyltransferase 1 (CARM1, also known as PRMT4), the most predominant ADMA methyltransferase, catalyzes the methylation of histone H3 at Arg 17 and 26, and the former is a major methylation site (Guccione and Richard, 2019). CARM1 plays important roles in autophagy, pluripotency of embryonic stem cells, regulation of RNA metabolism and ferroptosis (Shin et al., 2016; Suresh et al., 2021; Wang et al., 2018, 2022). Furthermore, it promotes breast cancer progression and metastasis in estrogen receptor α (ERα)-positive breast cancer (Peng et al., 2020; Wang et al., 2014). However, little is known about the functionality of CARM1 in TNBC.

The hypoxia-inducible factor-1 (HIF-1) can be stably expressed under hypoxic conditions and can bind to and activate specific genes in many mammalian cells (Burr et al., 2016). It consists of HIF1A or HIF2A, and HIF1B/ARNT (aryl hydrocarbon receptor nuclear translocator) subunits. Under normoxia, proline in HIF1A is converted into hydroxyproline by proline hydroxylase. This hydroxylated HIF1A can be recognized by the von Hippel-Lindau (VHL) protein, dependent on α-ketoglutarate-dependent dioxygenases, prolyl hydroxylases (PHD), and asparaginyl hydroxylase, factor-inhibiting HIF (FIH). Then, the ubiquitinase system can label and degrade hydroxylated HIF1A by the proteasome (Dey et al., 2020). On the other hand, under hypoxia, PHDs are inactive; therefore, HIF1A synthesizes and enters the nucleus to activate the expression of a large number of genes related to hypoxia adaptation, such as VEGFA (vascular endothelial growth factor A) and proteins that regulate the synthesis of EPO (erythropoietin) (Choudhry and Harris, 2018). Hypoxia is involved in many biological processes, including glycolysis, angiogenesis, cell proliferation, and apoptosis (Ceranski et al., 2023; Cowman and Koh, 2022; Madan et al., 2019).

In this study, we identified that CARM1 is a potential biomarker in TNBC and upregulated in multiple cancers. CARM1 cooperates with HIF1A to occupy the promoters of *CDK4*, *Cyclin D1*, *β-Catenin*, *HIF1A*, *MALAT1*, and *SIX1* and promote tumor progression in TNBC. Ellagic acid, a natural inhibitor of CARM1, decreases the expression of CDK4 to suppress the proliferation of TNBC. Our data indicate that CARM1 is a potential target and that ellagic acid is promising for the treatment of TNBC.

## Results

### Upregulation of CARM1 is correlated with breast cancer progression

**Figure 1.**
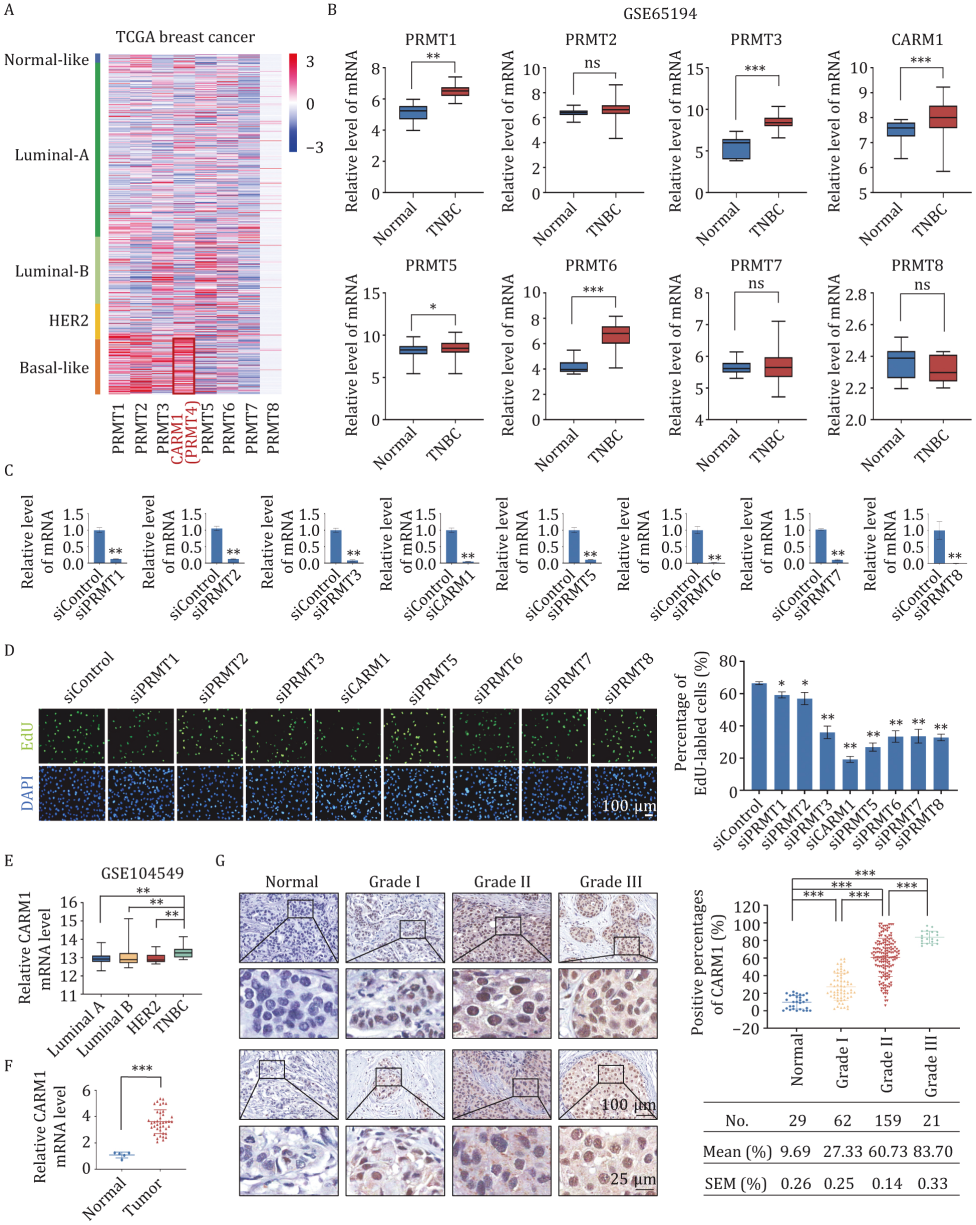
Upregulation of CARM1 is correlated with breast cancer progression. (A) Heatmap showing the gene expression status of the PRMT family in different subtypes of breast cancer according to TCGA. (B) Analysis of GSE65194 for the expression of PRMT family in normal or TNBC tissues (*P < 0.05, **P < 0.01, ***P < 0.001, ns, not significant; two-tailed unpaired *t*-test). (C) MDA-MB-231 cells were transfected with siControl or siRNAs targeting the PRMT family. Knockdown efficiencies were verified by RT-qPCR. (D) EdU cell proliferation assays of MDA-MB-231 cells transfected with siControl and siRNAs specifically targeting PRMT family members. Scale bar, 100 μm. (E) Analysis of GSE104549 for the expression of CARM1 in distinct subtypes of breast cancer (**P < 0.01; two-tailed unpaired *t*-test). (F) Detection of CARM1 in the breast cancer tissue cDNA array by RT-qPCR (***P < 0.001; two-tailed unpaired *t*-test). (G) Immunohistochemical staining of CARM1 in normal breast and breast tissues carcinomas (histological grades I, II, and III). The images represent fields under microscopy from two different cases in each grade. Positively stained nuclei in the grouped samples were calculated as percentages (***P < 0.001; two-tailed unpaired *t*-test). (C and D) Error bars represent the mean ± SD of three independent experiments (*P < 0.05, **P < 0.01; two-tailed unpaired *t*-test).

To explore the expression of PRMT family members in breast cancer, we analyzed published clinical data. From the GEO DataSets GSE42568, we found that PRMT1, PRMT2, and CARM1 were highly expressed in breast cancer cells (Fig. S1A). According to the Cancer Genome Atlas (TCGA) database, CARM1 was highly expressed in basal-like breast cancer (Fig. 1A). Additionally, the data in GSE65194 showed that PRMT1, PRMT3, CARM1, PRMT5, and PRMT6 were highly expressed in basal-like breast cancer (Fig. 1B). Then, we transfected MDA-MB-231 cells with siRNAs targeting the PRMT family and evaluated knockdown efficiencies at the mRNA (Fig. 1C) and protein levels (Fig. S1B). CARM1 depletion led to a significant decrease in the percentage of BrdU (EdU)-labeled cells compared with the control and other PRMT family members by an enhanced EdU incorporation assay (Fig. 1D). Furthermore, wound-healing assay results revealed that CARM1 knockdown resulted in a much lower migration rate in MDA-MB-231 cells (Fig. S1C). To further determine the expression of CARM1 in basal-like breast cancers, we analyzed published clinical data from GSE104549. We found that CARM1 expression was elevated in triple-negative breast cancers (Fig. 1E). Using the human cancer survey RT-qPCR gene expression panel in breast tissue, we observed that CARM1 was highly expressed in breast cancer samples compared with normal tissues (Fig. 1F).

To explore the role of CARM1 in breast cancer progression, immunohistochemical analysis of breast carcinoma samples and normal mammary tissues was performed (Fig. 1G). CARM1 was upregulated in breast cancer samples and positively correlated with histological grade. In conclusion, these data support our hypothesis that CARM1 promotes proliferation and migration in TNBC and that its upregulation is linked to breast cancer progression.

### CARM1 promotes proliferation, invasion, epithelial–mesenchymal transition (EMT), and stemness in TNBC

**Figure 2.**
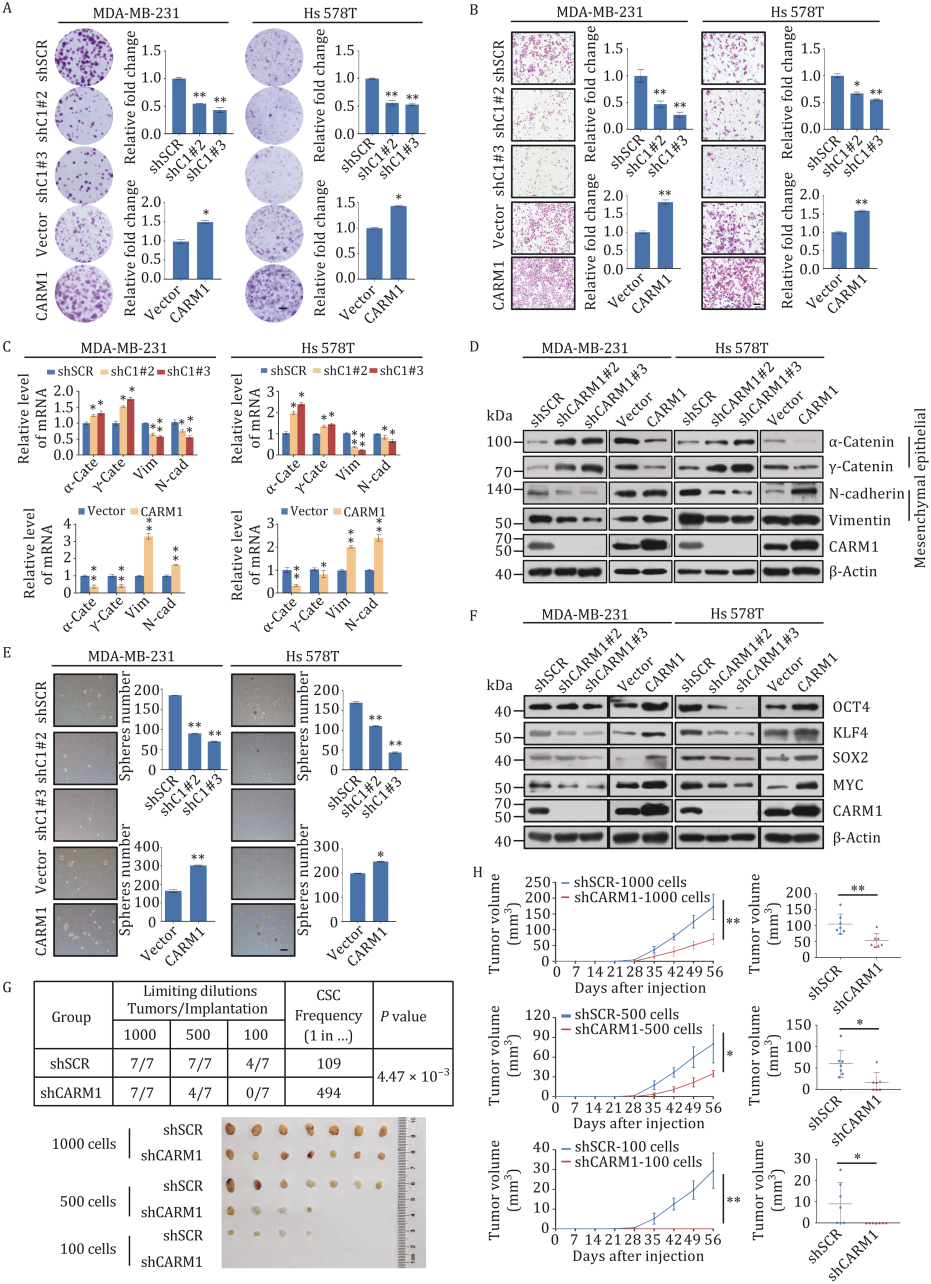
CARM1 promotes proliferation, invasion, epithelial–mesenchymal transition (EMT), and stemness in TNBC. (A) Representative photos of colony formation assays were performed on MDA-MB-231 and Hs 578T cells transfected with shSCR, two different CARM1 shRNAs, or vector, or FLAG-CARM1. Scale bar, 100 μm. C1, CARM1. (B) Cell invasion assays were carried out using Matrigel transwell ﬁlters in MDA-MB-231 and and Hs 578T cells. The images represent one field under microscopy in each group. Scale bar, 100 μm. C1, CARM1. (C and D) Expression of the indicated epithelial or mesenchymal markers was measured by RT-qPCR (C) and Western blot (D). α-cate, α-Catenin; γ-cate, γ-Catenin; Vim, Vimentin; N-cad, N-cadherin. (E) Representative images of sphere numbers in mammosphere assays. Scale bar, 100 μm. C1, CARM1. (F) Expression of the indicated stemness markers was measured by western blot. (G) MDA-MB-231 cells stably transfected with shSCR or shCARM1 were implanted into the fourth mammary fat pad of NOD SCID mice with various dilutions (*n* = 5). The frequency of CSCs was calculated using the online tool extreme limiting dilution analysis (ELDA). (H) The tumor growth curves of different numbers (1000, 500, and 100) of shSCR or shCARM1 cells were monitored during the specified time period. The tumor volume at the end point is shown on the right. (*P < 0.05, **P < 0.01; two-tailed unpaired *t*-test). (A–E) Error bars represent the mean ± SD of three independent experiments (*P < 0.05, **P < 0.01; two-tailed unpaired *t*-test).

To determine the role of CARM1 in TNBC, gain- and loss-of-function experiments of CARM1 were performed. For gain-of-function, breast cancer cells were infected with lentivirus delivering CARM1 CDS (coding sequence); and for loss-of-function, breast cancer cell lines with stably depleted CARM1 were generated using three different lentivirus-delivered shRNAs. The shRNAs led to a significant reduction in the expression of their target genes at both the mRNA and protein levels (Fig. S2A). Therefore, the two most efficient shRNAs were selected for subsequent experiments. To study the effect of CARM1 on proliferation, growth curves, and colony formation assays were performed (Figs. 2A and S2B). Compared with the control, CARM1 overexpression increased the proliferation rate, whereas its knockdown resulted in growth inhibition. To examine the role of CARM1 in the invasion and metastasis of TNBC, transwell assays were performed in MDA-MB-231 and Hs 578T cells (Fig. 2B). CARM1 overexpression increased cell invasion, whereas its knockdown by two separate shRNAs significantly decreased cell invasion. In addition, the expression of EMT markers at the mRNA and protein levels was detected in MDA-MB-231 and Hs 578T cells (Fig. 2C and 2D). The expression of epithelial markers, such as α-Catenin and γ-Catenin, increased, whereas that of certain mesenchymal markers, such as N-cadherin and Vimentin, decreased in CARM1-depleted cells. Meanwhile, the reverse results were observed in CARM1-overexpressing cells.

According to previous reports, CARM1 regulates pluripotency in early mouse embryos and is required to maintain pluripotency in embryonic stem cells (Goolam et al., 2016; Hupalowska et al., 2018). Thus, we explored the potential role of CARM1 in TNBC stemness using mammosphere formation assays (Figs. 2E and S2C). CARM1 knockdown significantly reduced the number and diameter of tumor spheres, whereas its overexpression resulted in formation of more spheres with larger diameters, suggesting that CARM1 promotes stemness in TNBC. The expression of stemness markers was also detected, and we found increased expression of OCT4, KLF4, SOX2, and MYC at both the mRNA and protein levels following CARM1 overexpression (Figs. S2D and 2F). Consistently, the expression of these markers decreased following CARM1 knockdown. In addition, various dilutions of MDA-MB-231 cells (1000, 500, and 100) stably transfected with control and shCARM1 lentiviruses were implanted into NOD SCID mice (Fig. 2G and 2H). The limited dilution of MDA-MB-231 cell transplantation resulted in a low frequency of tumor formation. However, after knockdown, tumor formation was greatly reduced. The control group produced tumors as few as 100 cells, whereas the CARM1-knockdown group did not form any tumors. Our data showed that CARM1 promotes carcinogenesis both *in vivo* and *in vitro*, indicating that CARM1 promotes the proliferation, invasion, EMT, and stemness potential of TNBC cells.

### Identiﬁcation of genome-wide transcription targets for CARM1

**Figure 3.**
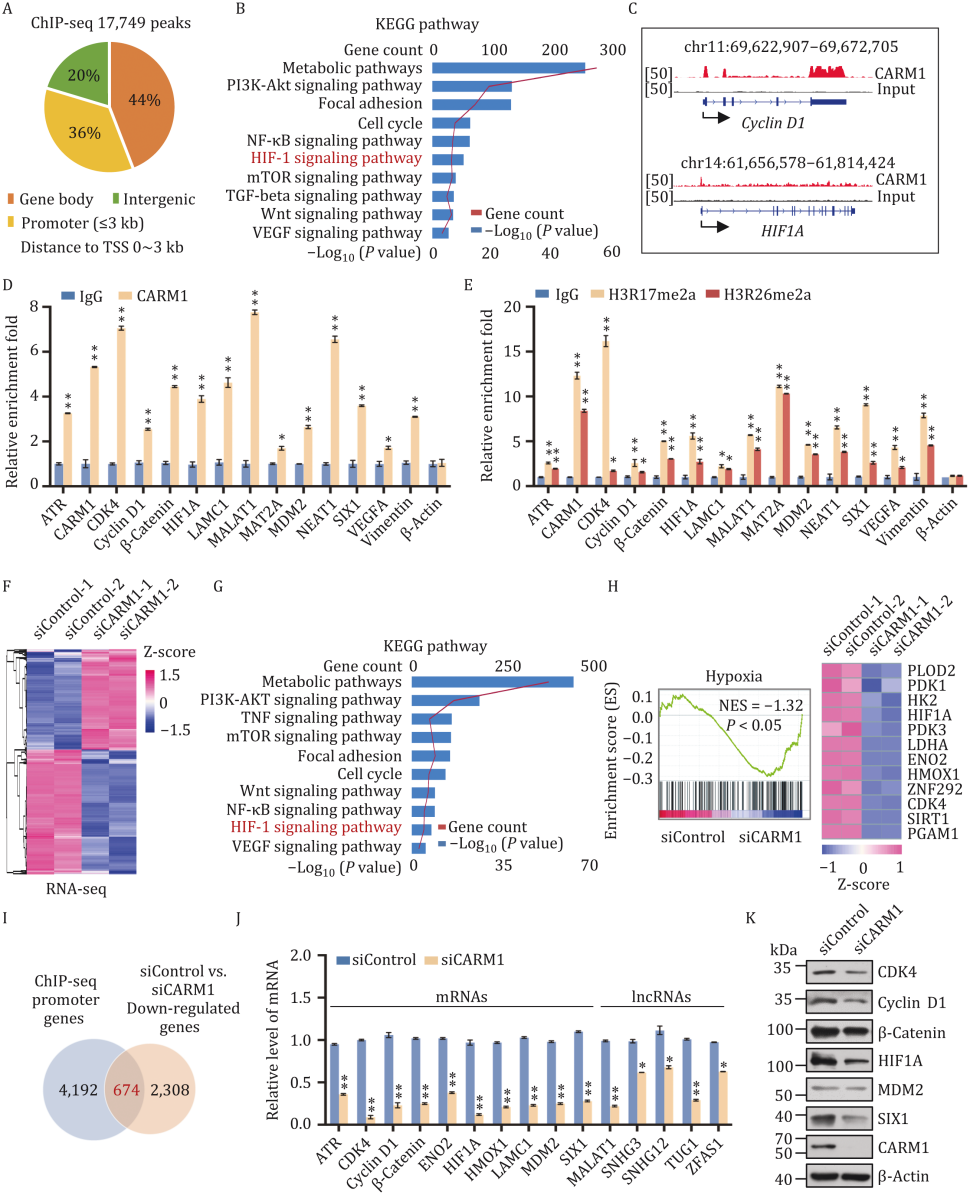
Identiﬁcation of genome-wide transcription targets for CARM1. (A) Genomic distribution of CARM1 sites. (B) KEGG pathway analysis of CARM1 ChIP-seq promoter genes arranged into functional groups. Data were analyzed using KOBAS website. (C) Binding proﬁles of CARM1 on representative target genes *Cyclin D1* and *HIF1A*. (D and E) qChIP veriﬁcation of the ChIP-seq results on the promoter of the indicated genes with antibodies against the indicated proteins in MDA-MB-231 cells. Data are presented as fold-change relative to that of β-Actin (negative control). (F) Heatmap representation of differentially expressed genes in control siRNA (siControl-1, siControl-2) and CARM1 knockdown (KD) (siCARM1-1, siCARM1-2) MDA-MB-231 cells. Two independent samples were used for the RNA-seq analysis. Data were analyzed using R package (v3.6.3). The rows show the *Z*-scores calculated for each group. (G) KEGG pathway analysis of the differentially expressed genes of CARM1 arranged into functional groups. (H) GSEA of RNA-seq data. NES: normalized enrichment score. (I) The promoter genes in ChIP-seq were overlapped with downregulated mRNAs in CARM1 mRNA-seq (*P* < 0.001). The overlapped gene numbers are shown in the venn diagram. (J) RT-qPCR experiments were performed to verify the results of RNA-seq. (K) Verification of RNA-seq results by Western blot. (D, E, and J) Error bars represent the mean ± SD of three independent experiments (*P < 0.05, **P < 0.01; two-tailed unpaired *t*-test).

To determine the genomic binding landscape of CARM1, we analyzed its genome-wide transcriptional targets using chromatin immunoprecipitation-based deep sequencing (ChIP-seq) in MDA-MB-231 cells with antibodies against CARM1. Following ChIP, CARM1-precipitated DNA was amplified using non-biased conditions, labeled, and sequenced on the Illumina HiSeq 2500 platform. A total of 17,749 CARM1-specific binding peaks and 4,866 unique promoter genes with a *P*-value cutoff of 10^−3^ were identified (Fig. 3A). Detailed ChIP-seq data are provided in GEO (GEO accession number: GSE171767). According to the results of the Kyoto Encyclopedia of Genes and Genomes (KEGG) pathway analysis, these genes participated in metabolic pathways, focal adhesion, cell cycle, HIF-1 signaling pathway, Wnt signaling pathway, and VEGF signaling pathway (Fig. 3B). Representative ChIP-seq peak data are shown in Fig. 3C. Quantitative ChIP (qChIP) analysis in MDA-MB-231 cells using specific antibodies against CARM1 on selected genes, including *ATR*, *CARM1*, *CDK4*, *Cyclin D1*, *β-Catenin*, *HIF1A*, *LAMC1*, *MALAT1*, *MAT2A*, *MDM2*, *NEAT1*, *SIX1*, *VEGFA*, and *Vimentin*, showed a strong enrichment of CARM1 on the promoters of these genes, thus validating our ChIP-seq results (Fig. 3D). Surprisingly, we found that the enrichment of CARM1 on the promoters of CARM1 were also increased, which indicated that CARM1 could activate itself and induce positive feedback. In addition, qChIP analysis with specific antibodies against H3R17me2a and H3R26me2a on selected genes was carried out, which showed that H3R17me2a and H3R26me2a occupied the target promoters (Fig. 3E). Our data further showed that these promoters are occupied by CARM1.

To investigate the molecular mechanism underlying the promotion of TNBC proliferation and migration by CARM1, we next carried out RNA sequencing experiments using control or siRNAs against CARM1 in MDA-MB-231 cells. Two independent controls and samples (siControl-1, siControl-2, and siCARM1-1, siCARM1-2) were used in these experiments. We found 2,386 upregulated genes and 2,982 downregulated genes in CARM1-deficient cells [|fold change (*FC*)| > 1.5, *P* < 0.001] (GEO accession number: GSE167394). The volcano plot and heatmap of differentially expressed genes (DEGs) are shown in Figs. S3A and 3F. According to the results of the KEGG pathway analysis, the potential targets of CARM1 are involved in various biological processes, including metabolic pathways, focal adhesion, cell cycle, Wnt signaling pathway, HIF-1 signaling pathway, and VEGF signaling pathway (Fig. 3G), which was consistent with that of ChIP-seq data. Subsequently, gene set enrichment analysis (GSEA) revealed that CARM1 promotes the hypoxia signaling pathway and EMT (Figs. 3H and S3B). The representative genes in these pathways such as CDK4, Cyclin D1, β-Catenin, HIF1A, and SIX1 were also marked in the volcano plot (Fig. S3A).

Interestingly, several hypoxia- or EMT- associated lncRNAs were found in our ChIP-seq data, including MALAT1 (also known as NEAT2) and TUG1. As previously reported, NEAT1 and MALAT1 are the main lncRNAs induced by hypoxia and contain HIF-binding sites close to their promoters. Compared with NEAT1, which was downregulated in the absence of HIF2A, MALAT1 was downregulated by both HIF1A and HIF2A siRNA (Choudhry et al., 2014, 2016). To verify the potential significance of these lncRNAs, lncRNA-seq was used to assess lncRNA expression levels. We found 160 upregulated and 141 downregulated lncRNAs in CARM1-deficient cells in comparison with the control (*FC* > 1.2, *P* < 0.001) (GEO accession number: GSE168121). A volcano plot of all the expressed lncRNAs is shown in Fig. S3C. Among them, MALAT1 was the most remarkable lncRNA that was downregulated in TNBC. In summary, our data demonstrated close relationships between CARM1, HIF1A, and MALAT1.

To identify the connections between ChIP-seq and RNA-seq, the promoter genes in ChIP-seq (*P* < 0.001) were overlapped with the mRNAs downregulated in CARM1 RNA-seq (|*FC*| > 1.5, *P* < 0.001) (Fig. 3I). To confirm the changes caused by CARM1 knockdown, putative target genes were validated using reverse transcription-quantitative polymerase chain reaction (RT-qPCR) in MDA-MB-231 cells (Fig. 3J) and verified by Western blot (Fig. 3K).

### CARM1 is physically associated and directly interacts with HIF1A

**Figure 4.**
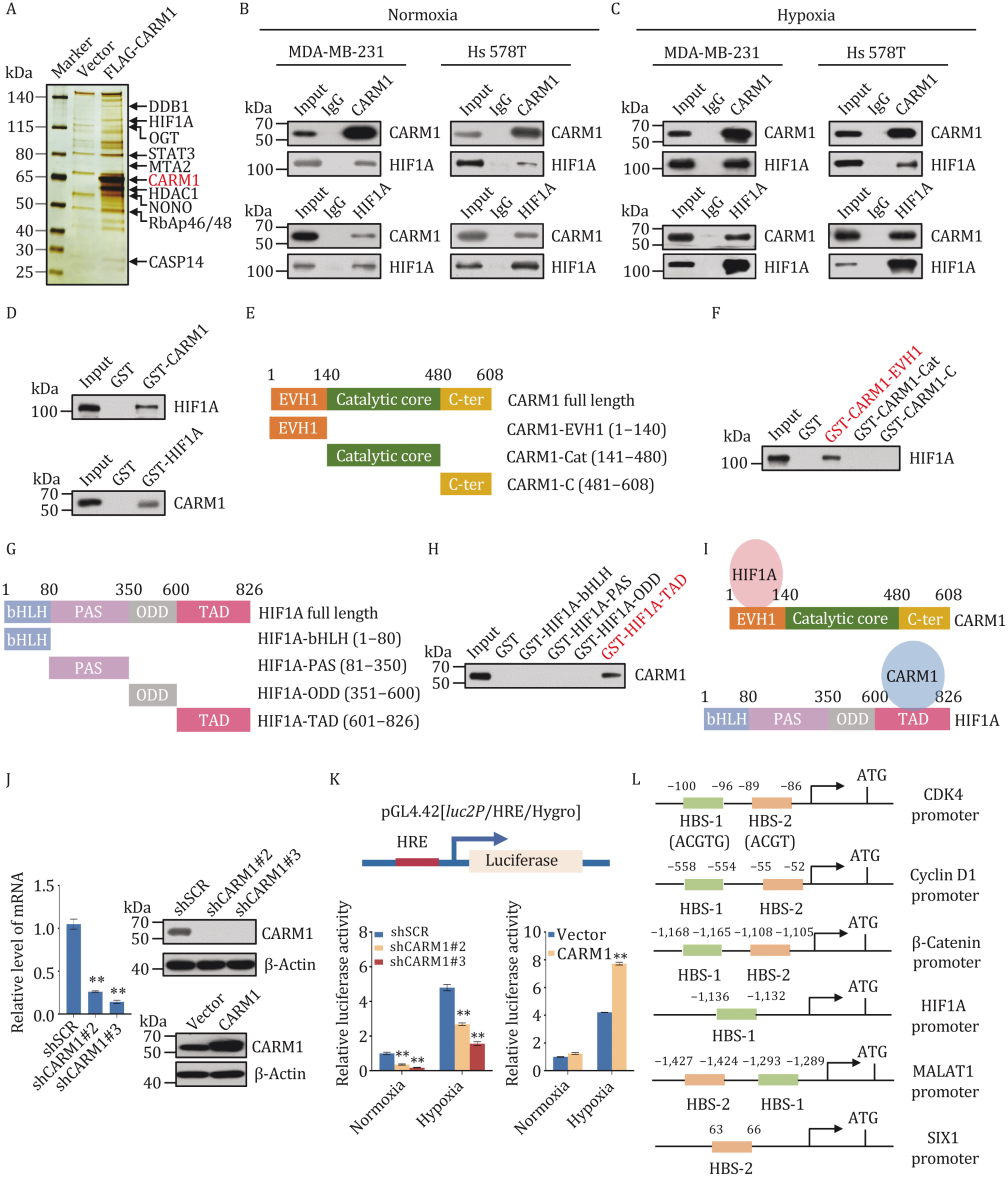
CARM1 is physically associated and directly interacts with HIF1A. (A) Immunoaffinity purification and mass spectrometry analysis of CARM1-associated proteins in MDA-MB-231 cells. (B and C) Association of CARM1 and HIF1A in MDA-MB-231 and Hs 578T cells under normoxia (B) or hypoxia (cells treated with 1% O_2_ for 24 h) (C) Whole cell lysates were prepared, and co-immunoprecipitation experiments were performed with antibodies against the indicated proteins. Immunocomplexes were then immunoblotted using antibodies against the indicated proteins. IgG served as the negative control. (D) Molecular interactions between CARM1 and HIF1A. GST pull-down assays using bacterially expressed GST-fused proteins and *in vitro* transcribed/translated proteins are shown. (E and F) Identification of essential domains required for the interaction with HIF1A of CARM1. GST pull-down experiments with bacterially expressed series of truncation vectors of CARM1 [EVH1, catalytic core and C-ter] to generate GST fusion proteins and *in vitro* transcribed/translated indicated proteins. (G and I) Identification of essential domains required for the interaction with CARM1 of HIF1A. GST pull-down experiments with bacterially expressed series of truncation vectors of HIF1A (bHLH, PAS, ODD, and TAD) to generate GST fusion proteins and *in vitro* transcribed/translated indicated proteins. (J) MDA-MB-231 cells were transfected with shSCR or two different shRNAs targeting CARM1 or vector or FLAG-CARM1. Efficiency was verified using RT-qPCR and Western blot. (K) HeLa cells stably infected with lentivirus were transfected with pGL4.42[luc2P/HRE/Hygro] plasmid and treated with 1% O_2_ for 24 h. Then, luciferase assays were performed. (L) Schematic representation of the promoter region of CDK4, Cyclin D1, β-Catenin, HIF1A, MALAT1, and SIX1. (J and K) Error bars represent the mean ± SD of three independent experiments (**P < 0.01; two-tailed unpaired *t*-test).

To better understand the mechanism of CARM1 in TNBC, we employed afﬁnity puriﬁcation and mass spectrometry (Table S4) to identify the proteins interacting with CARM1 and verified the results using Western blot (Figs. 4A and S4A). In these experiments, FLAG-tagged vector and CARM1 (FLAG-CARM1) were stably expressed in MDA-MB-231 cells. Whole cell extracts were prepared and subjected to affinity purification using an anti-FLAG affinity gel. Subsequent mass spectrometry analysis of the bound proteins revealed that CARM1 is physically associated with HIF1A.

To verify the interaction between CARM1 and HIF1A, cell lysates from MDA-MB-231, Hs 578T, and MDA-MB-468 were extracted under normoxia and hypoxia and subjected to co-immunoprecipitation (co-IP) experiments (Figs. 4B, 4C, S4B and S4C). This revealed that CARM1 and HIF1A immunoprecipitated with each other. While CARM1 and HIF2α did not interact with each other under normoxia or hypoxia conditions (Fig. S4D and S4E), even HIF2α activation is also known to be a strong oncogenic event (Zhuang et al., 2012). To further determine the molecular basis for the interaction between CARM1 and HIF1A, GST pull-down assays were conducted using a GST-fused CARM1 construct and *in vitro* transcribed/translated HIF1A and revealed that CARM1 interacts directly with HIF1A (Figs. 4D and S4F). Similar results were obtained from reciprocal GST pull-down experiments with GST-fused HIF1A and *in vitro* transcribed/translated CARM1. Additionally, GST pull-down assays with the GST-fused CARM1 EVH1 domain [1–140 amino acid (aa), CARM1-EVH1], catalytic core (141–480 aa, CARM1-Cat), and C-terminus (481–608 aa, CARM1-C) of CARM1 (Shishkova et al., 2017) and *in vitro* transcribed/translated HIF1A indicated that the EVH1 domain not catalytic core of CARM1 is responsible for its interaction with HIF1A (Figs. 4E, 4F, 4I and S4H), which suggested that CARM1 interacts with HIF1A not by its catalytic function. Consistently, the GST pull-down results showed that CARM1 with EVH1 domain deletion (141–608 aa, CARM1-∆EVH1) could not interact with HIF1A, and the methyltransferase inactive CARM1-R168A mutant (Genois et al., 2021) does not affect the interaction between CARM1 and HIF1A (Fig. S4G and S4H). In other words, the interaction is not in a manner of post-translational modification (PTM). Likewise, GST pull-down assays with the GST-fused HIF1A bHLH domain (1–80 aa, HIF1A-bHLH), PAS (81–350 aa, HIF1A-PAS), ODD (351–600 aa, HIF1A-ODD), and TAD (601–826 aa, HIF1A-TAD) of HIF1A and *in vitro* transcribed/translated CARM1 clarified that the TAD domain of HIF1A is responsible for its interaction with CARM1 (Figs. 4G–I and S4I). The TAD domain is reported to activate transcription of HIF1A when bound to DNA in complex with a β subunit (Altun et al., 2012; Kaelin, 2005; Shin et al., 2008; Yoo et al., 2006).

To further detect whether CARM1 can directly regulate the hypoxia signaling pathway, HeLa cells with no ERα were stably infected with CARM1 lentivirus carrying control shRNA (shSCR) or two different shRNAs targeting CARM1 or vector or FLAG-CARM1 (Giamas et al., 2009). The efficiencies are shown in Fig. 4J. Next, the cells were transfected with pGL4.42[l*uc2P*/HRE/Hygro] vector containing hypoxia response elements (HREs) to detect luciferase activity under normoxia and hypoxia (Fig. 4K). The results showed that CARM1 directly regulated the hypoxia signaling pathway. The promoters of *CDK4*, *Cyclin D1*, *β-Catenin*, *HIF1A*, *MALAT1*, and *SIX1* were then analyzed using the BIOBASE, which showed that they all have potential HIF-1 binding sequences (Kimura et al., 2001) (Fig. 4L). In conclusion, our results illustrated that CARM1 directly interacts with HIF1A and positively regulates its expression.

### HIF1A recruits CARM1 for transcriptional activation in TNBC

**Figure 5.**
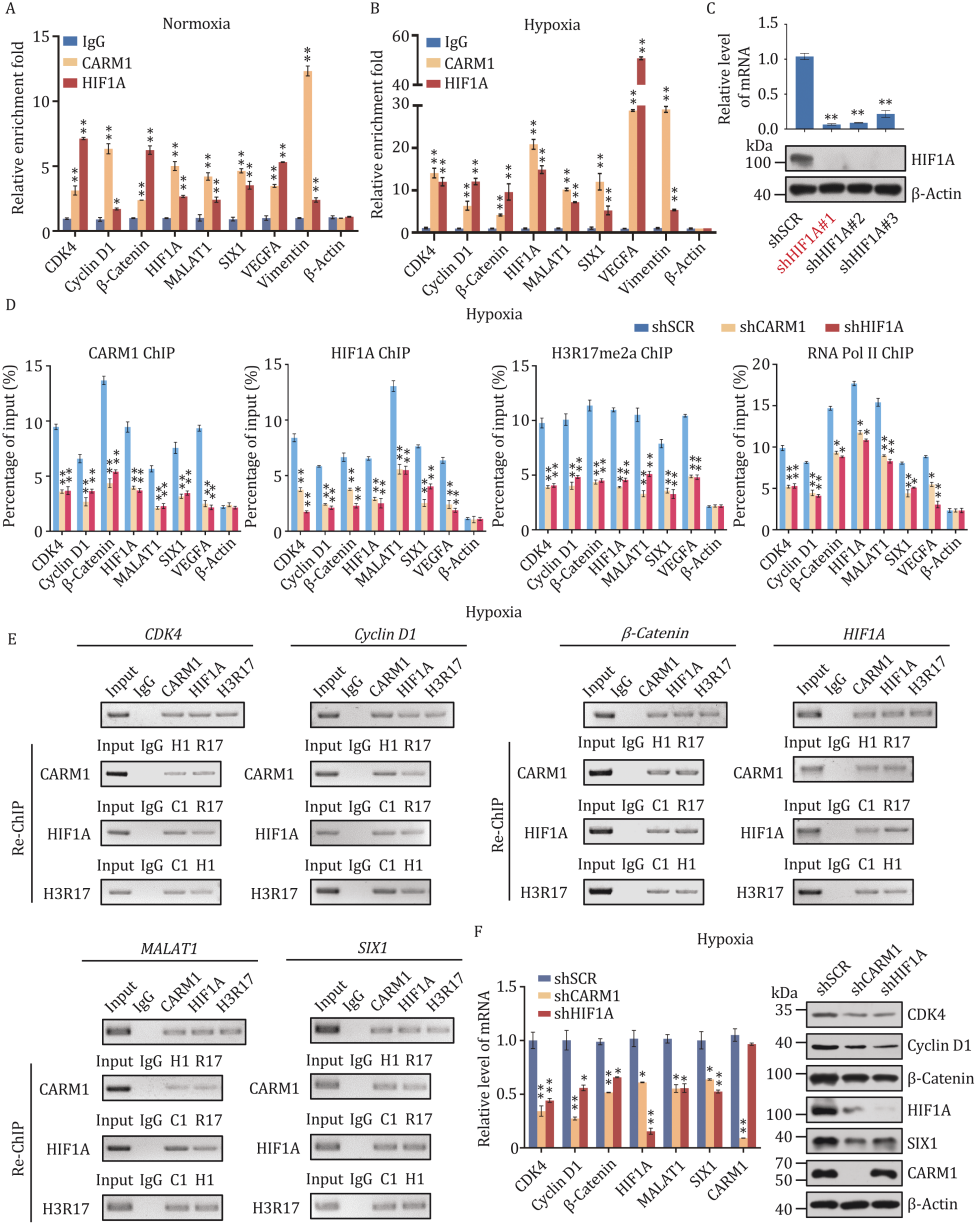
HIF1A recruits CARM1 for transcriptional activation in TNBC. (A and B) qChIP experiments with antibodies against IgG, CARM1, and HIF1A in MDA-MB-231 cells under normoxia (A) or hypoxia (1% O_2_ for 24 h) (B) with MALAT1 and VEGFA as positive controls. (C) MDA-MB-231 cells were transfected with shSCR and three different shRNAs targeting HIF1A. Knockdown efficiencies were verified by RT-qPCR and Western blot. The most efficient shHIF1A#1 was used for the subsequent experiments. (D) qChIP analysis of selected promoters in MDA-MB-231 cells stably transfected with control shRNA or shRNAs targeting CARM1 or HIF1A under hypoxia (1% O_2_ for 24 h) using the indicated antibodies. Data are presented as the fold-change relative to β-Actin, the negative control. (E) ChIP-Re-ChIP experiments were performed in MDA-MB-231 cells under hypoxia. C1, CARM1; H1, HIF1A; R17, H3R17me2a. (F) MDA-MB-231 cells stably infected with control shRNA or shRNAs targeting CARM1 or HIF1A treated with 1% O_2_ for 24 h, and the mRNA and protein levels of CDK4, Cyclin D1, β-Catenin, HIF1A, MALAT1, and SIX1 were measured and normalized to those of β-Actin. (A–D, and F) Error bars represent the mean ± SD of three independent experiments (*P < 0.05, **P < 0.01; two-tailed unpaired *t*-test).

HIF1A is a transcription factor acting to activate gene transcription (Majmundar et al., 2010). As described above, CARM1 is physically associated with HIF1A, suggesting the notion that HIF1A/CARM1 constitutes an activation complex functioning to promote gene transcription. To explore the functional significance of the physical association between HIF1A and CARM1, qChIP experiments were conducted with antibodies against CARM1 and HIF1A in MDA-MB-231 cells under normoxia and hypoxia, respectively, and *MALAT1* and *VEGFA* were used as positive controls. The results showed that CARM1 and HIF1A co-occupied these targets (Fig. 5A and 5B). For further exploration, MDA-MB-231 cells were stably infected with HIF1A lentivirus carrying shSCR or three different shRNAs targeting HIF1A, and the efficiency was tested at both the mRNA and protein levels. The most efficient one (marked in red) was chosen for subsequent experiments (Fig. 5C).

To test our hypothesis that CARM1 and HIF1A occupy the target promoters as a protein complex, MDA-MB-231 cells stably transfected with lentivirus carrying shSCR or shRNAs targeting CARM1 and HIF1A were used to conduct qChIP experiments under hypoxia as indicated target genes. CARM1 knockdown significantly decreased the binding of HIF1A to the promoters of *CDK4*, *Cyclin D1*, *β-Catenin*, *HIF1A*, *MALAT1*, *SIX1*, and *VEGFA*, and vice versa (Fig. 5D). Meanwhile, the H3R17me2a and RNA polymerase II (Pol II) levels of all tested target promoters were significantly reduced after the knockdown of CARM1 or HIF1A (Fig. 5D).

To further verify our hypothesis, sequential ChIP or ChIP-Re-ChIP assays were performed on six representative target genes, *CDK4*, *Cyclin D1*, *β-Catenin*, *HIF1A*, *MALAT1*, and *SIX1*. In these experiments, soluble chromatin was immunoprecipitated with antibodies against CARM1, HIF1A, and H3R17me2a. H3R17me2a was used as a positive control. The immunoprecipitates were subsequently re-immunoprecipitated using the appropriate antibodies. The results showed that, in precipitates, the *CDK4*, *Cyclin D1*, *β-Catenin*, *HIF1A*, *MALAT1*, and *SIX1* promoters that were immunoprecipitated with antibodies against CARM1 could be re-immunoprecipitated with antibodies against HIF1A or H3R17me2a (Fig. 5E). Similar results were obtained when an initial ChIP assay was performed with antibodies against HIF1A or H3R17me2a (Fig. 5E). These results support our hypothesis that HIF1A and CARM1 occupy the promoters of *CDK4*, *Cyclin D1*, *β-Catenin*, *HIF1A*, *MALAT1*, and *SIX1* as one protein complex. As expected, HIF1A and CARM1 deletion downregulated the expression of CDK4, Cyclin D1, β-Catenin, HIF1A, MALAT1, and SIX1 both at the mRNA and protein levels in MDA-MB-231 cells under hypoxia (Fig. 5F). Collectively, our results support that HIF1A and CARM1 as a whole bind to the target gene promoters, and mutually promote their recruitment and/or stabilization, and jointly play the function of transcription activation.

### CARM1 coordinates with HIF1A to promote the proliferation, invasion, and stemness in TNBC

**Figure 6.**
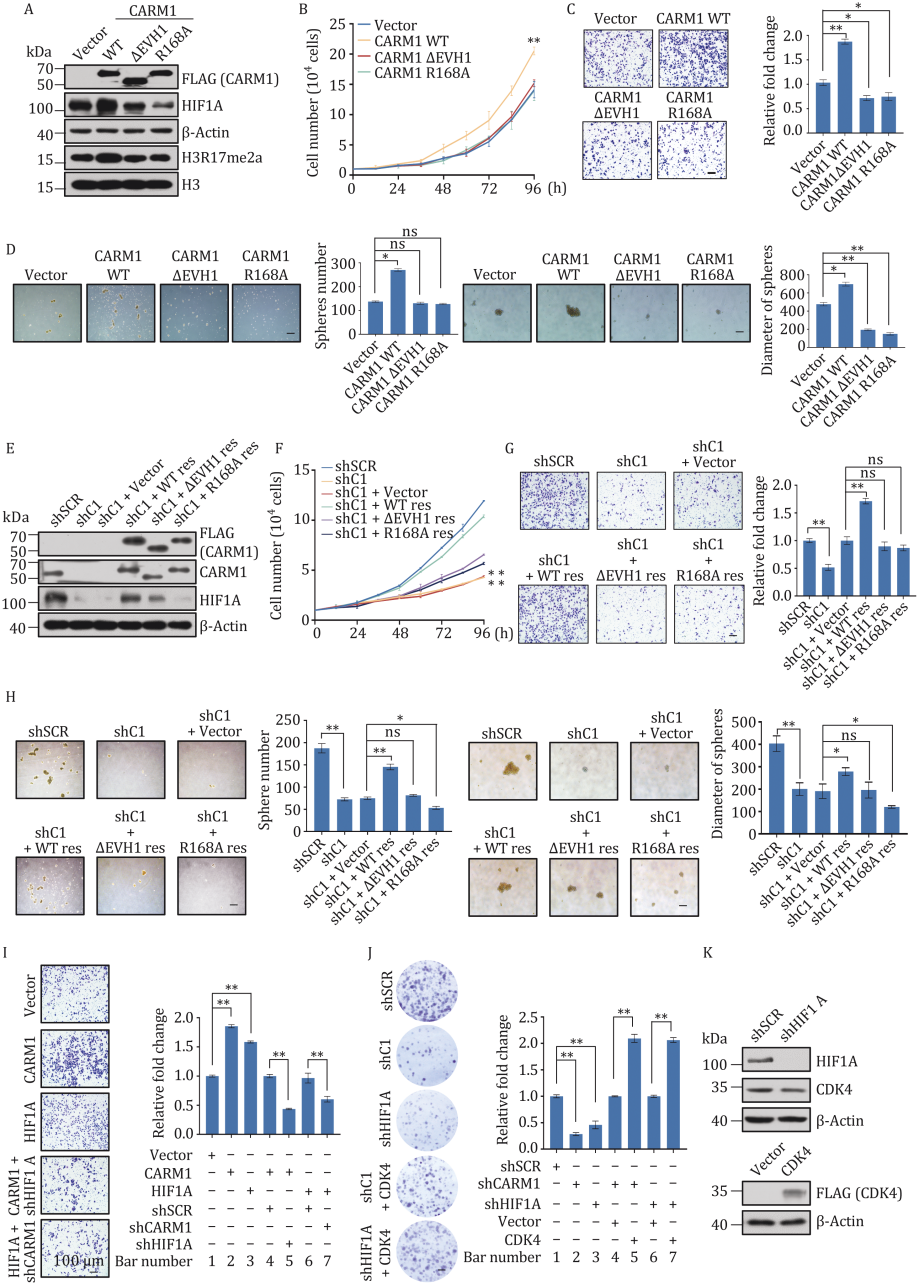
CARM1 coordinates with HIF1A to promote the proliferation, invasion, and stemness in TNBC. (A) Western blot analysis of the indicated proteins in MDA-MB-231 cells transfected with different lentiviruses. (B) Representative photos of growth curve assays performed on MDA-MB-231 cells transfected with vector, wild type CARM1 (CARM1 WT), EVH1 domain deletion (CARM1 ∆EVH1), or single point mutation R168A (CARM1 R168A) viruses. (C) Representative photos of transwell assays performed on MDA-MB-231 cells transfected with different lentiviruses. Scale bar, 100 μm. (D) Representative photos of mammosphere formation assays performed on MDA-MB-231 cells transfected with different lentiviruses. Scale bar, 100 μm. (E) Western blot of protein expression using antibodies against the indicated proteins in MDA-MB-231 cells transfected with different lentiviruses. (F) Representative photos of growth curve assays performed on MDA-MB-231 cells transfected with shSCR, shCARM1 (shC1), and shCARM1 together with vector, WT res, ∆EVH1 res, or R168A res. (G) Representative photos of transwell assays performed on MDA-MB-231 cells transfected with different lentiviruses. Scale bar, 100 μm. (H) Representative photos of mammosphere formation assays performed on MDA-MB-231 cells transfected with different lentiviruses. Scale bar, 100 μm. (I) CARM1 and HIF1A collaboratively enhanced the proliferation and invasion of breast cancer cells. MDA-MB-231 cells were stably transfected with CARM1, HIF1A, shRNAs, and expression vectors as indicated for the transwell assays. (J) Representative photos of colony formation assays performed on MDA-MB-231 cells transfected with the indicated speciﬁc shRNA and/or expression constructs. (K) Western blot was used to determine the protein expression in these cells using antibodies against the indicated proteins. (B–D, F–J) Error bars represent the mean ± SD of three independent experiments (*P < 0.05, **P < 0.01; two-tailed unpaired *t*-test).

To explore whether CARM1 promotes cancer development and migration depends on enzyme activity or transcriptional regulation, we designed the following viruses and transfected them in MDA-MB-231 cells: vector, wild type CARM1 (CARM1 WT), EVH1 domain deletion (CARM1 ∆EVH1), and single point mutation R168A (CARM1 R168A) viruses. The EVH1 domain is responsible for the binding of CARM1 and HIF1A, and R168A is the point mutation site of CARM1 enzyme activity region (Genois et al., 2021; Kim et al., 2010; Wang et al., 2016; Zhong et al., 2018). The efﬁciencies of these lentiviruses are shown in Fig. 6A. Then we used these cells to perform a growth curve experiment. Results showed increased cell numbers in cells transfected with CARM1 WT, whereas almost no change was observed in those without the EVH1 domain or R168A (Fig. 6B). Moreover, transwell assays showed that CARM1 WT promoted the invasion ability of MDA-MB-231 cells, whereas the loss of the EVH1 domain or R168A had little effect on cell invasion (Fig. 6C). Simultaneously, cells transfected with CARM1 WT had significantly increased numbers of spheres and larger diameters in mammosphere assays. However, the deletion of the EVH1 domain or R168A weakened the stemness-promoting ability of MDA-MB-231 cells (Fig. 6D). These results suggest that the EVH1 domain and R168A play important roles of CARM1 in promoting tumor progression. Subsequently, MDA-MB-231 cells were stably infected with shSCR, shCARM1, and shCARM1 together with vector, shRNA-resistant CARM1 (WT res), shRNA-resistant ∆EVH1 (∆EVH1 res) or R168A (R168A res). Western blot verified the efﬁciencies of the shRNAs used in these experiments (Fig. 6E). Growth curve assays were then performed. Knockdown of CARM1 decreased the cell numbers compared with the control, which could be significantly rescued by shRNA-resistant CARM1 and hardly rescued by shRNA-resistant ∆EVH1 or R168A (Fig. 6F). Furthermore, similar results were observed in the transwell and mammosphere assays (Fig. 6G and 6H). Our data showed that shRNA-resistant CARM1 rescued the expression of HIF1A in CARM1 knockdown cells, while both the HIF1A-binding mutants and R168A could hardly rescue the expression of HIF1A. These results indicate that CARM1 promotes proliferation, invasion, and stemness in a transcriptional regulation- and enzyme activity-dependent manner.

To determine whether HIF1A contributes to the function of CARM1 in TNBC, we ﬁrst analyzed the effect of gain-of-function and loss-of-function of CARM1 and HIF1A using transwell assays and colony formation in MDA-MB-231 cells. Stable HIF1A/CARM1 expression increased the number of invading cells compared with the control; however, the effect of their overexpression was diminished when cells were concomitantly knocked down by speciﬁc shRNA targeting HIF1A or CARM1 (Fig. 6I). Meanwhile, HIF1A/CARM1 knockdown decreased the number of colonies compared with controls, which was then rescued by CDK4 overexpression (Fig. 6J). Western blot veriﬁed the efﬁciencies of the lentiviruses used in these experiments (Fig. 6K). These results suggest that CARM1 and HIF1A are mutually dependent and collectively modulate the proliferation and invasion of MDA-MB-231 cells by activating CDK4 expression. Therefore, CARM1 and HIF1A jointly promote invasion and proliferation.

### CARM1 inhibitor ellagic acid suppresses proliferation and invasion in TNBC

**Figure 7.**
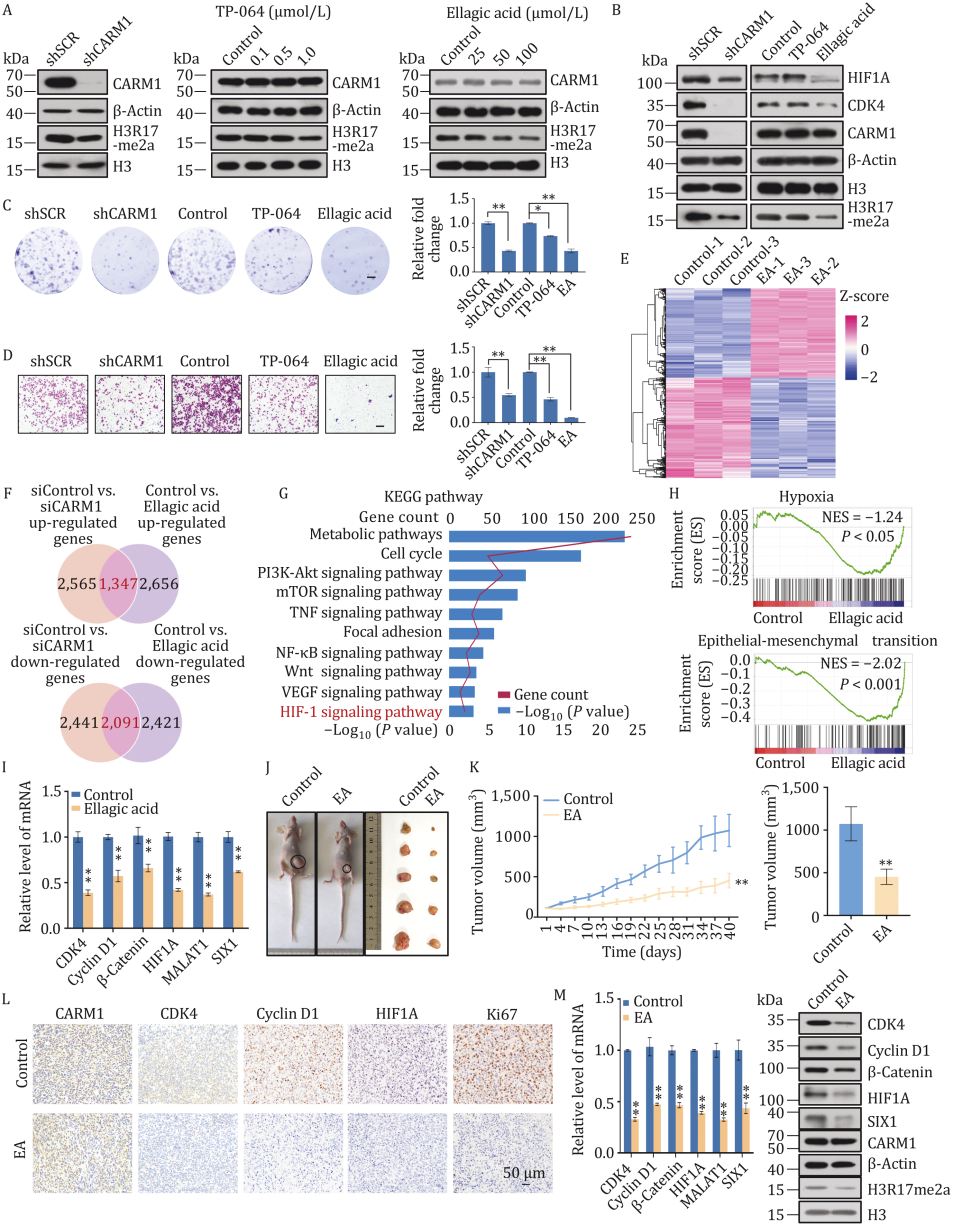
CARM1 inhibitor ellagic acid suppresses proliferation and invasion in TNBC. (A) Efficiencies of shRNA targeting CARM1, TP-064 (12 h), or ellagic acid (24 h) in MDA-MB-231 cells were evaluated by Western blot. (B) Western blot of indicated proteins in MDA-MB-231 cells treated with shRNA targeting CARM1, TP-064 (1 μmol/L, 12 h), or ellagic acid (100 μmol/L, 24 h). (C) Transwell assays of MDA-MB-231 cells. Scale bar, 100 μm. (D) Colony formation assays of MDA-MB-231 cells. Scale bar, 100 μm. (E) Heatmaps showing DEGs (|*FC*| > 1.2, *P* < 0.001) in the control and ellagic acid treatment of MDA-MB-231 cells. Three independent samples were used in RNA-seq analysis. Data were analyzed using R package (version 3.6.3). Rows show the *Z*-scores calculated for each group. EA, ellagic acid. (F) The up-regulated genes in CARM1 RNA-seq (|*FC*| > 1.2, *P* < 0.001) were overlapped with those in RNA-seq cells treated with ellagic acid (|*FC*| > 1.2, *P* < 0.001). The down-regulated genes in CARM1 RNA-seq seq (|*FC*| > 1.2, *P* < 0.001) were overlapped with those in RNA-seq cells treated with ellagic acid (|*FC*| > 1.2, *P* < 0.001). The numbers of overlapped genes are shown in Venn diagram. (G) KEGG pathway analysis of the DEGs of the overlapped genes arranged into functional groups (|*FC*| > 1.2, *P* < 0.001). Data were analyzed using KOBAS website. (H) GSEA of the overlapped data (*P* < 0.001). (I) Verification of RNA-seq results by RT-qPCR. (J) BALB/c nude mice bearing MDA-MB-231 cells were treated with the control or ellagic acid (50 mg/kg/day) by gavage. Representative mice with MDA-MB-231 xenografts (left) and tumors dissected from the control or ellagic acid-treated mice (right) are shown (*n* = 5). (K) Tumor growth of xenograft nude mice treated with the control or ellagic acid were measured every three days (**P < 0.01; two-tailed unpaired *t*-test). (L) Tumor tissues were prepared for IHC detection with speciﬁc antibodies against CARM1, CDK4, Cyclin D1, HIF1A, and Ki67. (M) RT-qPCR (left) and Western blot analysis (right) were performed. (C, D, I, and M) Each bar represents the mean ± SD of triplicate experiments (*P < 0.05, **P < 0.01; two-tailed unpaired *t*-test).

For further investigation, two CARM1 inhibitors were chosen from previous reports and their efficiencies were tested compared with shRNAs against CARM1 in MDA-MB-231 cells (Fig. 7A). Ellagic acid is a polyphenol dilactone that can be isolated from crude pomegranate extract. It is a CARM1 site-specific inhibitor that blocks CARM1-mediated H3R17 dimethylation (Selvi et al., 2010; Shin et al., 2016). On the other hand, TP-064 is a potent and selective chemical probe for CARM1 that effectively inhibits its methyltransferase activity (Nakayama et al., 2018; Zhong et al., 2018). Next, we detected the expression of HIF1A and CDK4 through these inhibitors. Surprisingly, CDK4 expression was almost knocked out following CARM1 knockdown, with the efficiency of ellagic acid greater than that of TP-064 (Fig. 7B). Subsequently, transwell and colony formation assays showed that cells treated with ellagic acid had lower proliferation rates and invasion capacity compared with shCARM1 and TP-064 (Fig. 7C and 7D). After treatment with TP-064 or ellagic acid, MDA-MB-231 cells with CARM1 stably depleted did not show a decrease in MDA-MB-231 cell invasiveness (Fig. S5A and S5B), indicating that CARM1 deficiency significantly inhibited the invasive potential of MDA-MB-231 cells and reduced the efficiency of TP-064 or ellagic acid.

Ellagic acid has been reported to inhibit tumor growth in breast cancer, glioblastoma, and pancreatic cancer both *in vitro* and *in vivo*. Additionally, it can exert anti-angiogenic effects by regulating VEGF, VEGFR, and HIF1A in breast cancer (Wang et al., 2012, 2017a, 2017b; Zhao et al., 2013). Moreover, ellagic acid intervention did not have a clear effect on the interaction between CARM1 and HIF1A (Fig. S5C). Thus, we investigated the effects of ellagic acid on TNBC cells using RNA-seq experiments (GEO accession number: GSE171618) in MDA-MB-231 cells treated with DMSO and ellagic acid. A heatmap is shown in Fig. 7E. We found 4,003 upregulated and 4,512 downregulated genes (|*FC*| > 1.2, *P* < 0.001). Based on the overlap between the genes up-/down-regulated in CARM1-knockdown RNA-seq and those in ellagic acid-treated RNA-seq (Fig. 7F), we found 1,347 upregulated and 2,091 downregulated genes, including *CDK4*, *Cyclin D1*, *β-Catenin*, *HIF1A*, *MALAT1*, and *SIX1.* Moreover, KEGG analysis of overlapped genes revealed that these genes were involved in metabolic pathways, cell cycle, focal adhesion, Wnt signaling pathway, VEGF signaling pathway, and HIF-1 signaling pathway (Fig. 7G). GSEA showed that these targets were associated with hypoxia and EMT (Fig. 7H). These analyses were consistent with the results of CARM1-knockdown RNA-seq data. RT-qPCR results of representative genes showed that the expression of *CDK4*, *Cyclin D1*, *β-Catenin*, *HIF1A*, *MALAT1*, and *SIX1* decreased in ellagic acid-treated cells, thus verifying that ellagic acid regulates these genes (Fig. 7I). These suggest that ellagic acid plays an anticancer role as a CARM1 inhibitor in TNBC.

To explore the function of ellagic acid *in vivo*, MDA-MB-231 cells were orthotopically implanted into the abdominal mammary fat pad of BALB/c nude mice. After tumor formation, the mice were randomly divided into two groups: the control group and the ellagic acid group (50 mg/kg/day); each group contained five mice. The tumor volume of mice treated with ellagic acid through gavage was smaller than that of the control group, indicating that ellagic acid suppressed tumor growth (Fig. 7J and 7K). Meanwhile, the body weights of tumor-bearing mice showed no significant change (Fig. S5D). Tissue sections from the control and ellagic acid groups were subjected to hematoxylin and eosin (H&E) staining. No obvious pathological changes were observed in the main organs, including the heart, liver, spleen, lung, and kidney (Fig. S5E), indicating that ellagic acid did not cause serious or adverse changes *in vivo*. Moreover, the influence of ellagic acid on tumor tissues was determined using IHC, RT-qPCR, and Western blot (Fig. 7L and 7M). The expression of CDK4, Cyclin D1, HIF1A, and Ki67 was decreased in the ellagic acid-treated tissues compared with the control group and CARM1 expression was no change. Ellagic acid also inhibited xenograft proliferation by suppressing the mRNA and protein levels of CDK4, Cyclin D1, β-Catenin, HIF1A, MALAT1, and SIX1. Overall, we demonstrated that ellagic acid inhibits tumorigenesis of TNBC cells both *in vitro* and *in vivo*.

### CARM1 is upregulated in multiple cancers and is a potential cancer biomarker

**Figure 8.**
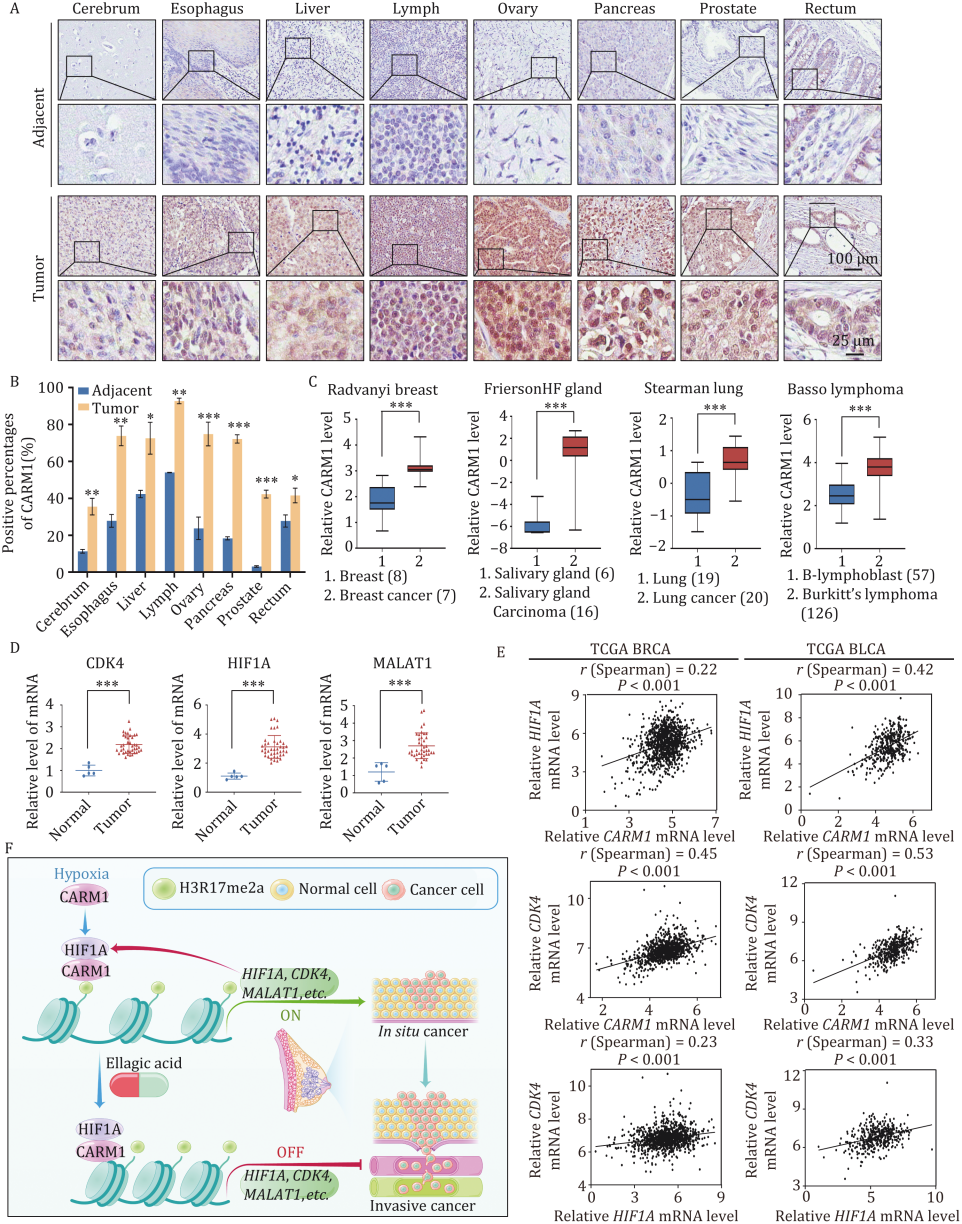
CARM1 is upregulated in multiple cancers and is a potential cancer biomarker. (A) Representative images of immunostained paired samples of cerebrum, esophagus, liver, lymph, ovary, pancreas, prostate, and rectum cancer tissues vs. adjacent normal tissue samples. (B) Positively stained nuclei were calculated as percentages (*P < 0.05, **P < 0.01, ***P < 0.001; two-tailed unpaired *t*-test). (C) CARM1 expression in multiple cancer microarray datasets available from Oncomine (***P < 0.001; two-tailed unpaired *t*-test). (D) Detection of *CDK4*, *HIF1A*, and *MALAT1* in the breast cancer tissue cDNA array by RT-qPCR (Each bar represents the mean ± SD of triplicate experiments (***P < 0.001; two-tailed unpaired *t*-test). (E) Correlation analysis of public TCGA datasets for the expression of CARM1, HIF1A, and CDK4 in BRCA and BLCA. (F) Proposed mechanism of the coordinated role of CARM1 and HIF1A in TNBC tumorigenesis.

To clarify whether CARM1 exerts tumorigenic effect on other cancers, we analyzed carcinoma samples from cerebrum, esophagus, liver, lymph, ovary, pancreas, prostate, and rectum cancer patients (*n* ≥ 15 for each cancer type paired with adjacent normal tissues). Tissue microarray analysis revealed the signiﬁcant upregulation of CARM1 expression in carcinomas from multiple tissues compared with adjacent normal tissues (Fig. 8A and 8B). Moreover, datasets analysis from Oncomine showed that CARM1 expression was significantly related to many carcinomas, such as breast cancer, salivary gland carcinoma, lung cancer, and Burkitt’s lymphoma (Fig. 8C). Thus, CARM1 is upregulated in multiple carcinomas and may be a potential cancer biomarker.

To investigate the relationship between CARM1 and CDK4/HIF1A/MALAT1, breast cancer tissue cDNA arrays were used to detect the expression of *CDK4*, *HIF1A*, and *MALAT1* in clinical samples. Compared with that in normal tissues, the expression of *CDK4*, *HIF1A*, and *MALAT1* were upregulated in the breast cancer samples (Fig. 8D), which was consistent with the results of CARM1 (Fig. 1F). Subsequently, correlation analysis of breast cancer in TCGA indicated that the expression of CARM1 is positively correlated with that of HIF1A and CDK4. Meanwhile, the expression of HIF1A was positively correlated with that of CDK4 (Fig. 8D). Similar results were obtained for bladder urothelial carcinoma (BLCA), cervical squamous cell carcinoma and endocervical adenocarcinoma (CESC), and esophageal carcinoma (ESCA) based on the TCGA database (Figs. 8E and S6). This supports our findings that CDK4 is transcriptionally regulated by CARM1 and HIF1A. In conclusion, CARM1 is a potential cancer biomarker and CARM1, HIF1A, and CDK4 are positively correlated in multiple cancers. The proposed mechanism of the coordinated role of CARM1 and HIF1A in TNBC tumorigenesis are described in Fig. 8F.

## Discussion

In this study, we revealed that CARM1 promotes carcinogenesis and metastasis in TNBC and is a potential biomarker for multiple cancers. CARM1 directly interacts with HIF1A and co-occupies the promoters of *CDK4*, *Cyclin D1*, *β-Catenin*, *HIF1A*, *MALAT1*, and *SIX1* to transactivate their expression. Moreover, we showed that ellagic acid, a natural inhibitor of CARM1, suppressed proliferation and invasion in TNBC and reduced CDK4 expression. Thus, we expect that CARM1 and ellagic acid have good potential as therapy target and treatment for TNBC, respectively.

As previously reported, CARM1 enhances breast cancer progression and metastasis in ERα-positive breast cancer and is a co-activator of ERα. In this study, CARM1 was highly expressed in TNBC and formed a coordinated complex with HIF1A to regulate downstream target genes. We focused on TNBC because it is more malignant than other breast cancer subtypes and has no effective therapies. Our research demonstrated that CARM1 is an important target for TNBC. Ellagic acid, its inhibitor, is harmless and a natural polyphenol health product. Our results show that ellagic acid (50 mg/kg/day) can effectively inhibit the occurrence, and development of TNBC in mice. According to the dose extrapolation between species recommended by FDA (Nair et al., 2018), the estimated dose for an adult (60 kg) is about 220 mg/day. In other words, patients may take 500 mg capsules of pomegranate extract containing 90% ellagic acid, such as the dose used in one Clinical Trial of ellagic acid on metabolic syndrome components (Clinical Trial NCT04011618). It is suggested that the development of ellagic acid-related products may have important clinical application value in the adjuvant treatment of TNBC.

The hypoxia pathway is related to metastasis, metabolism, angiogenesis, and stemness in various cancer types (de Heer et al., 2020; Eales et al., 2016; Rankin and Giaccia, 2016; Vadde et al., 2017). We focused on the role of hypoxia in TNBC. CARM1 and HIF1A form a complex that transcriptionally activates downstream target genes and promotes the occurrence and development of TNBC. *HIF1A* is not only the target gene of CARM1, but also an indispensable partner in promotion of TNBC carcinogenesis and metastasis. In the absence of HIF1A, the role of CARM1 in tumorigenesis is weakened. A previous report showed that CDK1 and CDK2 regulate the lysosomal degradation of HIF1A to promote cell-cycle progression (Hubbi et al., 2014; Zheng et al., 2019). Here, we found that CDK4 is a new target of HIF1A, which may provide new insights into cell cycle and hypoxia signaling pathways.

Under hypoxia, CARM1 is recruited by HIF1A and transcriptionally activates downstream target genes, such as *CDK4*, *Cyclin D1*, *β-Catenin*, *MALAT1*, and *SIX1*. Interestingly, HIF1A activate itself, forming a positive feedback. Our results suggest that the HIF1A/CARM1-CDK4/Cyclin D1-cell cycle checkpoint axis is responsive to hypoxic conditions. The overexpression of CDK4 can compensate for the decreased proliferation caused by CARM1- and HIF1A-knockdown. A previous study showed that CARM1 promotes tumorigenesis via the pGSK3β/β*-*Catenin/cyclin D1 signaling pathway in osteosarcoma (Li et al., 2017). The accumulation of *β-*Catenin may result in increased cyclin D1 level, which could then affect the cell cycle. Therefore, the HIF1A/CARM1-β-Catenin/cyclin D1 signaling pathway may also exist in TNBC. The relationship between β-Catenin and cyclin D1 will be explored in the future. Moreover, earlier studies have suggested that HIF1A regulates glycolysis (Chen et al., 2019; Peek et al., 2017; Weng et al., 2020). Our study consistently demonstrated that HIF1A/CARM1 may regulate glycolysis and metabolism by upregulating SIX1, a transcriptional factor related to the Warburg effect. However, the underlying mechanisms remain to be elucidated.

Remarkably, we also found that CARM1 was positively related to TUG1 and MALAT1. TUG1, which is abundant in human endothelial cells, is correlated with cell migration, proliferation, invasion, and EMT (Chen and Shen, 2020). On the other hand, MALAT1 plays a significant role in various cancer types (Hosseini et al., 2017). Hypoxic stress enhances the level of MALAT1 and MALAT1 has been reported as a target of HIF1A (Amodio et al., 2018; Lorenzen and Thum, 2016), which was also verified in this study. Thus, there is a certain connection between CARM1, HIF1A, and MALAT1, but the specific mechanism requires further study.

Ellagic acid, a polyphenol lactone widely found in various fruits and nuts, is a natural inhibitor of CARM1. Our results indicated that the inhibition of CARM1 suppressed the expression of CDK4. Similarly, ellagic acid inhibits the expression of CDK4, which can replace shRNA targeting CARM1. The direct inhibition of CDK4 by ellagic acid suppressed breast cancer proliferation. Atezolizumab plus nab-paclitaxel is used as the first-line treatment for unresectable, locally advanced, or metastatic TNBC (Schmid et al., 2020). Although CDK4/6 inhibitors for the treatment of metastatic hormone receptor-positive breast cancer have been studied (Gao et al., 2020; Spring et al., 2020), there are only a few studies on their therapeutic effect in TNBC. A study showed that trilaciclib (a selective, intravenously administered CDK4/6 inhibitor), together with gemcitabine and carboplatin chemotherapy, improved the overall survival of patients with metastatic TNBC (Tan et al., 2019). Our study suggests that ellagic acid may replace CDK4 inhibitors to inhibit carcinogenesis and treat TNBC through oral administration, it can be considered as a novel potential candidate for TNBC treatment, and it is worthy of further research and investigations.

Furthermore, ellagic acid has an antioxidant function and is related to reactive oxygen species (ROS) (Salimi et al., 2015; Zhang et al., 2014). Hypoxia results in increased ROS production via the electron transport chain (Fuhrmann and Brüne, 2017; Semenza, 2017; Smith et al., 2017). The genome is divided separately under hypoxia because there are no additional resources to consume. The genome is certainly not well replicated and assembled. In other words, the tumor mutation burden is too heavy. Our work suggests that ellagic acid can improve the vicious cycle of the cell cycle, which is abnormally activated by hypoxia.

This study has certain limitations. *In vitro* and *in vivo* experiments have confirmed the effect of ellagic acid on TNBC, but we have not conducted clinical trials. We will continue to explore the effects of ellagic acid on female TNBC from clinical trials. We hope that ellagic acid can be used clinically and solve the problem of difficult radical treatment of TNBC.

Overall, we confirmed that CARM1 is a key factor in promoting the occurrence and development of TNBC. It forms a complex with HIF1A to transactivate CDK4. The inhibition of CDK4 expression by ellagic acid inhibits the proliferation of breast cancer, so ellagic acid is expected to replace CDK4 inhibitors in the treatment of TNBC. Our findings paved the way for a better understanding of the mechanism of CARM1 function in TNBC.

## Materials and methods

### Antibodies and reagents

The following antibodies were used: anti-PRMT1 (2449S), anti-CARM1 (12495S), anti-PRMT6 (14641S), anti-KLF4 (12173S), anti-CDK4 (12790S), anti-Rpb1 NTD (14958S) (Cell Signaling Technology, Danvers, MA, USA); anti-FLAG (F1408), anti-Vimentin (V6630), and anti-β-Actin (A1978) (Sigma-Aldrich, St. Louis, MO, USA); anti-PRMT2 (ab154154), anti-PRMT3 (ab191562), anti-PRMT5 (ab109451), anti-PRMT8 (ab168134), anti-OCT4 (ab19857), anti-SOX2 (ab97959), anti-MYC (ab32072), anti-HIF1A (ab2185, ab51608), anti-H3R17me2a (ab8284), anti-H3R26me2a (ab194679), anti-H3 (ab1791) (Abcam, Cambridge, UK); anti-CARM1 (NB200-342), anti-PRMT7 (NBP2-19939) (Novus Biologicals, Littleton, Colorado, USA); anti-α-Catenin (610193), anti-γ-Catenin (610253), anti-N-cadherin (610920), anti-β-Catenin (610153) (BD Biosciences, Franklin Lakes, NJ, USA); anti-CDK4 (66950-1-Ig),anti-SIX1 (10709-1-AP) (Proteintech, Rocky Hill, NJ, USA); and anti-Cyclin D1 (0407-27), anti-MDM2 (ER1902-14) (Huabio, Hangzhou, China). Protein A/G Sepharose CL-4B beads were purchased from Amersham Biosciences (Amersham, UK), and the protease inhibitor cocktail was from Roche Applied Science (Basel, Switzerland). The siRNAs used in this study were esiRNAs from Sigma-Aldrich. The catalog are as follows: PRMT1, EHU115751; PRMT2, EHU134801; PRMT3, EHU083191; CARM1, EHU066371; PRMT5, EHU065921; PRMT6, EHU125311; PRMT7, EHU157661; PRMT8, EHU133921. The shRNAs were obtained from GenePharma Co. Ltd. (Shanghai, China). The human cancer survey RT-qPCR gene expression panels were from Origene (catalog number: BCRT102). TP-064 was purchased from Tocris (catalog number: 6008). Ellagic acid was from Cayman Chemical Co. (Ann Arbor, MI, USA).

### Cell culture and transfection

All cell lines were obtained from the American Type Culture Collection and authenticated in 2018 using short tandem repeat analysis. Hs578T and HeLa cells were cultured in Dulbecco’s modiﬁed Eagle’s medium (DMEM) in a humidified incubator equilibrated with 5% CO_2_ at 37°C, whereas MDA-MB-231 and MDA-MB-468 cells were maintained in L-15 medium without CO_2_. All media were supplemented with 10% fetal bovine serum, 100 units/mL penicillin, and 100 mg/mL streptomycin. For all hypoxic conditions, cells were placed in a modulator incubator chamber (Billups-Rothenberg, San Diego, California, USA) flushed with 1% O_2_. Transfections were carried out using Lipofectamine® RNAiMAX Reagent (Invitrogen) following the manufacturer’s instructions. Each experiment was performed in triplicate. For RNAi experiments, at least three independent shRNA sequences were tested for each gene, and the two with the highest efficiency were used. The shRNA sequences used in this study are listed in Table S1.

### Lentiviral production and infection

Recombinant lentiviruses expressing control shRNA (shSCR), shCARM1, shHIF1A, and shCDK4 were constructed by Shanghai GenePharma. The shRNA sequences used are listed in Table S1. The lentiviruses expressing empty vector and GFP-tagged CARM1 were constructed by Shanghai GenePharma Co., Ltd. (Shanghai, China). Concentrated viruses were used to infect 5 × 10^5^ cells in a 60 mm dish with 8 μg/mL polybrene. Stably expressing MDA-MB-231 or Hs 578T cells were screened with 2 μg/mL puromycin for 48 h. The infected cells were then subjected to sorting target expression.

### Reverse transcription-quantitative polymerase chain reaction (RT-qPCR)

Total RNA was extracted using TRIzol reagent according to the manufacturer’s instructions (Invitrogen). Potential DNA contamination was avoided using RNase-free DNase treatment (Promega, Madison, WI, USA). cDNA was prepared using MMLV reverse transcriptase (Promega). The relative quantitation of gene expression was carried out on the ABI PRISM 7500 sequence detection system (Applied Biosystems, Foster City, CA, USA), which measures real-time SYBR green ﬂuorescence, and calculated using the comparative Ct method (2^−△△^Ct) with β-Actin as an internal control. This experiment was independently performed at least thrice. The primer sequences used are shown in Table S2.

### 5-Ethynyl-20-deoxyuridine (EdU) assays

MDA-MB-231 cells after siRNAs transfected were seeded into 6-well plates, allowed to adhere overnight, and incubated in a conditioned medium from the EdU kit. To detect cell proliferation, they were subjected to the EdU assay according to the manufacturer’s instructions.

### Wound-healing assays

MDA-MB-231 cells after treatment were seeded into 6-well plates, and wounds were created using a 200 μL pipette tip. After washing with PBS to remove debris, the cells were visualized under a light microscope, and images were taken every 6 h. The relative migration rate of the cells was measured using Image J. The assays were independently performed at least three times.

### Tissue specimens and immunohistochemistry

We used IHC method to detect the expression of CARM1 antibody in breast cancer/multiple organ tumor and marginal tissue microarray. This part was largely completed by Xi’an Alena and Servicebio Biotechnology Ltd., Co. Steps are as follows: samples were frozen in liquid nitrogen immediately after surgical removal and maintained at −80°C until analysis. Samples were fixed in 4% paraformaldehyde (Sigma-Aldrich) at 4°C overnight, embedded in paraffin, sectioned (8 μm), and placed onto Superfrost-Plus Slides. They were stained with 3,3ʹ-diaminobenzidine (DAB) and monitored microscopically.

### Colony formation assays

MDA-MB-231 and Hs 578T cells were seeded on a fresh 6-well plate at a density of 1000 cells/well and cultured in a complete medium. After 10–14 days, the cells were fixed in methanol and stained with 0.1% crystal violet. The number of colonies was counted manually.

### Cell invasion assays

Transwell chamber filters (Chemicon Incorporation, Temecula, CA, USA) were coated with Matrigel. After lentiviral infection, MDA-MB-231 cells in serum-free L-15 medium were seeded into the upper chamber well (2 × 10^4^ cells) containing 500 μL of L-15 medium with 10% fetal bovine serum and incubated at 37°C for 18 h. Cells on the upper side of the membrane were removed using cotton swabs, whereas those on the other side were stained and counted. Three high-powered fields were counted for each membrane.

### Mammosphere culture

A total of 5,000 cells were plated in 6-well ultra-low attachment plates in serum-free DMEM-F12 supplemented with 0.4% BSA, 20 ng/mL bFGF, 10 ng/mL EGF, and 5 µg/mL insulin. Mammospheres were calculated after 14 days.

### Immunoprecipitation and Western blot

For immunoprecipitation assays, cells were washed with cold phosphate-buffered saline (PBS) and RIPA buffer supplemented with proteinase and phosphatase inhibitor cocktails for 30 min at 4°C. The cell lysates were centrifuged for 10 min at 13,000 rpm at 4°C to remove cellular debris. Next, 500 μg of cellular extract was incubated with the appropriate specific antibodies or normal rabbit/mouse immunoglobin G (IgG) on a rotator at 4°C overnight with constant rotation, followed by the addition of protein A/G Sepharose beads and incubation for 2 h at 4°C. The beads were washed five times with cell lysis buffer (50 mmol/L Tris-HCl at pH 7.4, 150 mmol/L NaCl, 1 mmol/L EDTA, 0.5% NP-40, 0.25% sodium deoxycholate, and protease inhibitor mixture). The immune complexes were subjected to sodium dodecyl sulfate–polyacrylamide gel electrophoresis (SDS-PAGE), followed by immunoblotting with secondary antibodies. Immunodetection was performed using enhanced chemiluminescence (ECL System, Thermo Fisher Scientific, Waltham, MA, USA) according to the manufacturer’s instructions.

### RNA sequencing (RNA-seq) and lncRNA sequencing (lncRNA-seq)

Total RNA (1 mg) was extracted and purified using oligo (dT)-attached magnetic beads, and RNA quality was assessed using an Agilent 2100 Bioanalyzer. Next, mRNA molecules were fragmented into small pieces using a fragmentation reagent, after which random hexamer-primed reverse transcription (RT) was performed to generate first-strand cDNA and double-stranded cDNA. The synthesized cDNA was subjected to end-repair and then 3ʹ-adenylated. Adapters were ligated to the ends of the cDNA fragments, and adapter-ligated libraries were generated by performing PCR with Illumina PE primers. The resulting cDNA libraries were applied onto an Illumina flow cell for cluster generation (TruSeq Cluster Generation Kit V.5) and sequenced. Genes with an *FPKM* > 0 in all samples were retained for further analysis. The mRNA-seq of CARM1 was used control or siRNAs against CARM1 in MDA-MB-231 cells. Two independent controls and samples (siControl-1, siControl-2, and siCARM1-1, siCARM1-2) were used in these experiments. DEGs between each group (*P* < 0.001 and fold-change > 1.5) were identified. The RNA-seq of ellagic acid was performed in MDA-MB-231 cells treated with DMSO and ellagic acid. Three independent controls and samples (Control-1, Control-2, Control-3, and EA-1, EA-2, EA-3) were used in these experiments. DEGs between each group (*P* < 0.001 and fold-change > 1.2) were identified.

For lncRNA-seq, the extracted total RNA was removed from ribosomal rRNA using a biotin-labeled specific probe (Ribo-Zero™ rRNA Removal Kit). After purification, the RNA is fragmented under a certain temperature and ionic environment. One-stranded cDNA is then synthesized using random primers and reverse transcriptase in the TruSeq® Stranded kit, followed by DNA polymerase I and RNaseH to synthesize double-stranded cDNA. During cDNA double-strand synthesis, the RNA template is removed and dTTP is replaced by dUTP. The involvement of dUTP prevents the second strand of the cDNA from being amplified in the subsequent procedure because the polymerase cannot cross the dUTP site on the template during extension. The double-stranded cDNA product was then subjected to “A” base addition and linker ligation. The ligation product will be amplified and purified to obtain the final cDNA library. Finally, the constructed sequencing library is sequenced on Illumina HiSeq platform by BGI Corporation (Shenzhen, China). LncRNAs with an *FPKM* > 0 in all samples were retained for further analysis. The lncRNA-seq of CARM1 was used control or siRNAs against CARM1 in MDA-MB-231 cells. One independent control and one sample (siControl and siCARM1) were used in these experiments. DEGs between each group (*FC* > 1.2, *P* < 0.001) were identiﬁed.

### Chromatin immunoprecipitation (ChIP), quantitative ChIP (qChIP), Re-ChIP, and ChIP-based deep sequencing (ChIP-seq)

ChIP experiments were performed in MDA-MB-231 cells as previously described (Wang et al., 2009). Briefly, 1 × 10^7^ cells were cross-linked with 1% formaldehyde, sonicated, pre-cleared, and incubated with 2–3 µg antibody for each reaction. The complexes were washed five times with low- and high-salt buffers, and DNA was purified using a QIAquick PCR Purification Kit. qRT-PCR was performed using the TransStart Top Green qPCR Supermix (TransGen Biotech, Shanghai, China). For Re-ChIP assays, bead eluates from the first immunoprecipitation were incubated with 20 mmol/L dithiothreitol (DTT) at 37°C for 30 min and diluted at a ratio of 1:50 in ChIP dilution buffer (1% Triton X-100, 2 mmol/L EDTA, 150 mmol/L NaCl, and 20 mmol/L Tris-HCl at pH 8.1), followed by re-immunoprecipitation with secondary antibodies. The final elution step was performed using 1% SDS in Tris-EDTA buffer (pH 8.0). Then, the obtained immunoprecipitation samples were subjected to PCR and agarose gel electrophoresis, and the bands were inverted to black for easy identification using Image lab software. The bands meant that these proteins were enriched on the promoter of the target genes. For ChIP-seq, a quantified 10 ng of DNA was resolved using an Agilent Technologies 2100 Bioanalyzer, with 50–250 bp fractions extracted and subjected to end-repair and 3ʹ- adenylation. Adapter-ligated libraries were amplified, purified, and selected using an Agencourt AMPure XP-Medium kit; the final library was composed of single-stranded circular DNA. In-depth whole-genome DNA sequencing was performed by the CapitalBio Corporation (Beijing, China). Sequencing data acquired from the Illumina analysis pipeline were compared with unmasked human reference genome hg38 (UCSC GRCh38) using ELAND (Illumina, San Diego, CA, USA). Peaks were called using Model-based Analysis of ChIP-Seq (MACS), following input filtering. ChIP seeker was used to analyze the genomic distribution of CARM1-binding sites. The primer sequences used are listed in Table S3.

### Immunopurification and mass spectrometry

Lysates from MDA-MB-231 cells stably transfected with vector and FLAG-CARM1 were injected into an equilibrated FLAG column. The column was then washed and eluted with FLAG peptide (Sigma-Aldrich). Fractions of the bed volume were collected, resolved by SDS-PAGE, and silver stained. The gel bands were then subjected to LC-MS/MS sequencing and analysis.

### Glutathione S-transferase (GST) pull-down experiments

GST-fused constructs were expressed in BL21 *Escherichia coli*. *In vitro* transcription and translation experiments were performed with rabbit reticulocyte lysate (TNT Systems, Promega) according to the manufacturer’s instructions. For GST pull-down assays, approximately 5 μg of the appropriate GST fusion proteins with 30 μL of glutathione–sepharose beads was incubated with 5–8 μL of *in vitro* transcribed/translated products in binding buffer (75 mmol/L NaCl, 50 mmol/L HEPES at pH 7.9) at 4°C for 2 h in the presence of the protease inhibitor mixture. The beads were washed five times with the binding buffer, resuspended in 30 μL of 2× SDS-PAGE loading buffer, and detected by Western blot.

### Luciferase reporter assays

HeLa cells stably infected with lentivirus were transfected with the pGL4.42 [luc2P/HRE/Hygro] plasmid (Promega, Madison, WI, USA) and treated with 1% O_2_ for 24 h. Luciferase activity was measured using a dual luciferase kit (Promega, Madison, WI, USA) according to the manufacturer’s protocol. Each experiment was performed in triplicate.

### Mouse xenograft models

To study stemness, MDA-MB-231 cells stably infected with lentiviruses carrying shSCR or shCARM1 were inoculated into the abdominal mammary fat pad of NOD SCID mice with various dilutions (1 × 10^3^, 5 × 10^2^, or 1 × 10^2^ cells; *n* = 7). The frequency of CSCs was calculated using the online tool Extreme Limiting Dilution Analysis (ELDA) (Hu and Smyth, 2009).

For the xenograft study of ellagic acid, MDA-MB-231 cells were inoculated into the left abdominal mammary fat pad (3 × 10^6^ cells) of 6-week-old female BALB/c nude mice to establish breast cancer xenografts. After tumor formation, the mice were randomly divided into two groups (*n* = 5 each): the control and the ellagic acid-treatment groups. The treatment group was administered with ellagic acid daily through gavage (50 mg/kg/day). Tumor volume and body weight were measured every 3 days. At the end of the experiment, all mice were imaged and euthanized. Tumor xenografts were dissected for final volume and wet weight measurements, immunohistochemical staining analysis, RT-qPCR, and Western blot. For toxicological analysis, deparafﬁnized normal tissue sections, including the heart, liver, spleen, lung, and kidney were stained with hematoxylin and eosin (H&E).

## Statistics

Data are reported as the mean ± SD of triplicate experiments, unless otherwise noted. Comparisons were performed using two-tailed unpaired *t*-tests. The correlation coefficients were calculated using the GraphPad Prism 7. Breast tumor datasets were downloaded from GEO DataSets (Ivhsina; GEO: GSE42568, GSE104549, and GSE19804).

## Supplementary data

The online version contains supplementary material available at https://doi.org/10.1093/procel/pwae010.

pwae010_suppl_Supplementary_Materials

## Data Availability

The data generated in this study are publicly available in Gene Expression Omnibus (GEO) at GSE171767 (ChIP-seq of CARM1), GSE167394 (mRNA-seq of CARM1), GSE168121 (lncRNA-seq of CARM1) and GSE171618 (RNA-seq of ellagic acid).
