# Supplementary material for: CARM1 drives triple-negative breast cancer progression by coordinating with HIF1A
Source: Protein Cell. 2024 Mar 13;15(10):744–65. doi: 10.1093/procel/pwae010 (PMC11443453; doi:10.1093/procel/pwae010)
Supplement: pwae010_suppl_Supplementary_Materials [file pwae010_suppl_supplementary_materials.pdf]

Supplementary File 1

CARM1 drives triple-negative breast cancer progression by coordinating with HIF1A

Dandan Feng[1,2,‡], Jie Gao[3,‡], Ruiqiong Liu[4,‡], Wei Liu[2], Tianyang Gao[2], Yunkai Yang[1], Die Zhang[1], Tianshu Yang[5], Xin Yin[5], Hefen Yu[5], Wei Huang[5,*], and Yan Wang[1,2,5,*]

Contents:

Supplementary Figures 1-6

Supplementary Figure 1. Upregulation of CARM1 is correlated with breast cancer progression. (A) Analysis of GSE42568 for the expression of PRMT family in normal or breast cancer tissues (*$P < 0.05$, **$P < 0.01$, ***$P < 0.001$, ns, not significant; two-tailed unpaired *t*-test). (B) MDA-MB-231 cells were transfected with control siRNA (siControl) or siRNAs targeting the PRMT family. Knockdown efficiencies were verified by western blot. (C) Wound-healing assays of MDA-MB-231 cells transfected with siControl and siRNAs specifically targeting PRMT family members. Scale bar, 100 μm. Error bars represent the mean ± SD of three independent experiments (*$P < 0.05$, **$P < 0.01$; two-tailed unpaired *t*-test).

Supplementary Figure 2. CARM1 promotes proliferation, invasion, epithelial-mesenchymal transition (EMT), and stemness in TNBC. (A) MDA-MB-231 and Hs 578T cells were transfected with control shRNA (shSCR) and three different shRNAs targeting CARM1, respectively. Knockdown efficiencies were verified by RT-qPCR and western blot. (B) Growth curve analysis was performed on MDA-MB-231 and Hs 578T cells transfected with shSCR, two different CARM1 shRNAs or transfected with vector or FLAG-CARM1. (C) Representative images of sphere diameters in mammosphere assays. Scale bar, 100 μm. (D) Expression of the indicated stemness markers was measured by RT-qPCR. (A–D) Error bars represent the mean ± SD of three independent experiments (*$P < 0.05$ and **$P < 0.01$; two-tailed unpaired *t*-test).

Supplementary Figure 3. Identification of genome-wide transcription targets for CARM1. (A) Volcano plots of all expressed genes show the differentially expressed genes

with fold-change > 1.5 and $P < 0.001$ (Bioinformatic analysis was performed using the OmicStudio tools). (B) GSEA of RNA-seq data. NES: normalized enrichment score. (C) Volcano plots of all expressed genes show the differentially expressed genes with fold-change > 1.2 and $P < 0.001$.

Supplementary Figure 4. CARM1 is physically associated and directly interacts with HIF1A. (A) Western blot analysis of the identified proteins in the purified fractions, using antibodies against the identified proteins. (B and C) Co-IP of CARM1 and HIF1A in MDA-MB-468 under normoxia (B) or hypoxia (cells treated with 1% O_2 for 24 h) (C). IgG served as the negative control. (D and E) Association of CARM1 and HIF2A in MDA-MB-231 and Hs 578T under normoxia (D) or hypoxia (cells treated with 1% O_2 for 24 h) (E). IgG served as the negative control. (F) Recombinant GST-CARM1 and GST-HIF1A proteins were detected by staining with Coomassie blue. (G) Identification of essential domains required for the interaction with HIF1A of CARM1. GST pull-down experiments with bacterially expressed series of truncation vectors of CARM1 [ΔEVH1, and R168A] to generate GST fusion proteins and in vitro transcribed/translated indicated proteins. (H and I) Recombinant GST proteins were detected by staining with Coomassie blue.

Supplementary Figure 5. CARM1 inhibitor ellagic acid suppresses proliferation and invasion in TNBC. (A) Transwell assays for shSCR, and CARM1-knockdown MDA-MB-231 cells treated with TP-064, or DMSO. Scale bar, 100 μm. Error bars represent the mean ± SD of three independent experiments (**$P < 0.01$, ns, not significant; two-tailed unpaired *t*-test). (B) Transwell assays for shSCR, and CARM1-knockdown MDA-MB-231 cells

treated with ellagic acid, or DMSO. Scale bar, 100 μm. EA, ellagic acid. Error bars represent the mean ± SD of three independent experiments (* $P < 0.05$ and ** $P < 0.01$; two-tailed unpaired *t*-test). (C) Lysates from MDA-MB-231 cells with or without Ellagic acid treatment immunoprecipitated with control IgG or an anti-CARM1 antibody, or an anti-HIF1A antibody, followed by immunoblotting with indicated antibodies. (D) The body weight of xenograft nude mice treated with the control or ellagic acid were measured every three days. Each bar represents the mean ± SD of triplicate experiments (two-tailed unpaired *t*-test). (E) Tissue sections from the control and ellagic acid groups were embedded in paraffin and stained by hematoxylin and eosin (H&E) for toxicological analysis.

Supplementary Figure 6. CARM1 is upregulated in multiple cancers and is a potential cancer biomarker. Correlation analysis of public TCGA datasets for the expression of CARM1, HIF1A, and CDK4 in cervical squamous cell carcinoma and endocervical adenocarcinoma (CESC), and esophageal carcinoma (ESCA) based on the TCGA database.

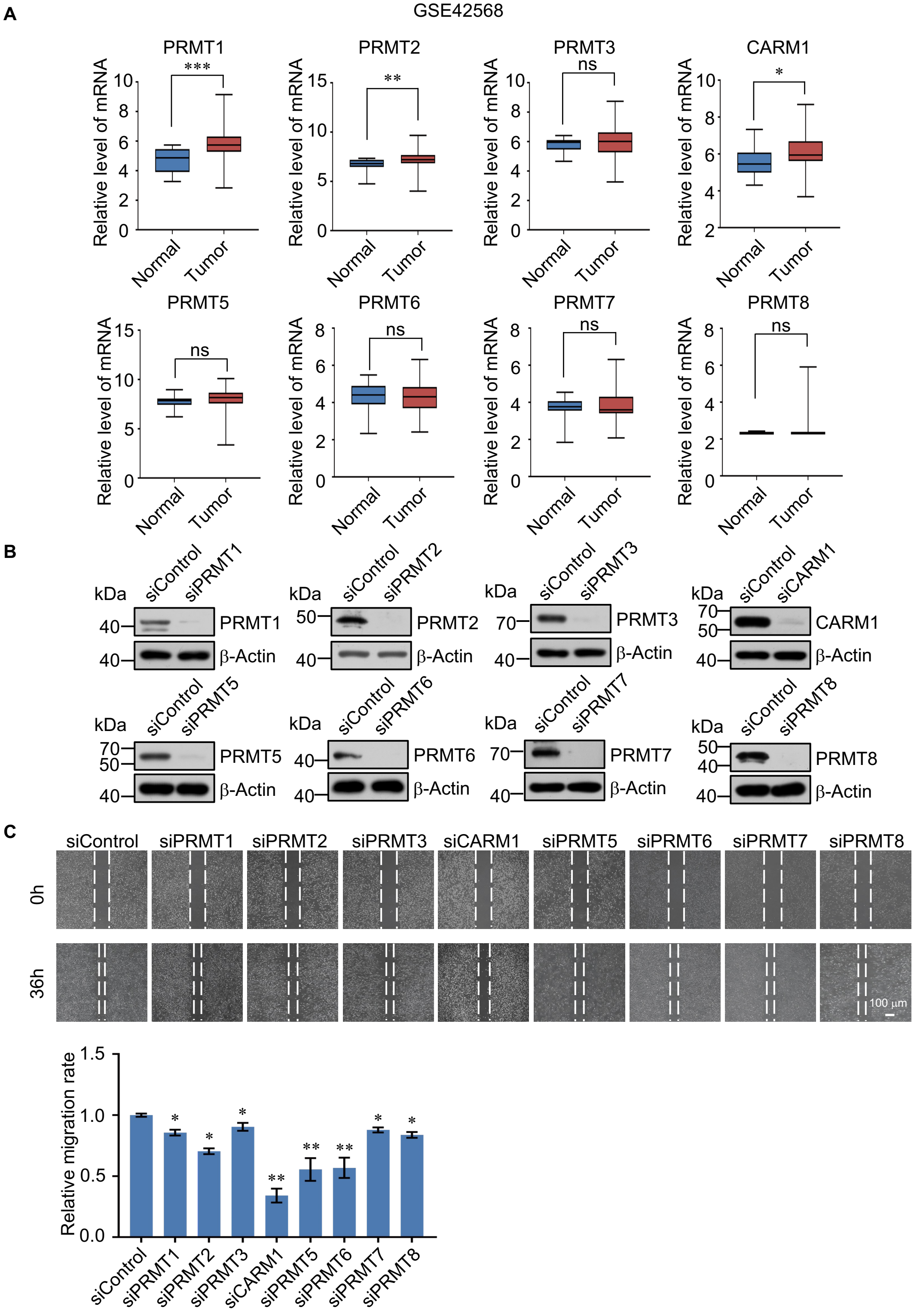
A
GSE42568
PRMT1
PRMT2
PRMT3
CARM1
PRMT5
PRMT6
PRMT7
PRMT8
***
**
ns
*
Relative level of mRNA
Normal
Tumor
B
kDa
siControl
siPRMT1
siPRMT2
siPRMT3
siCARM1
siPRMT5
siPRMT6
siPRMT7
siPRMT8
β-Actin
C
0h
36h
100 μm
Relative migration rate

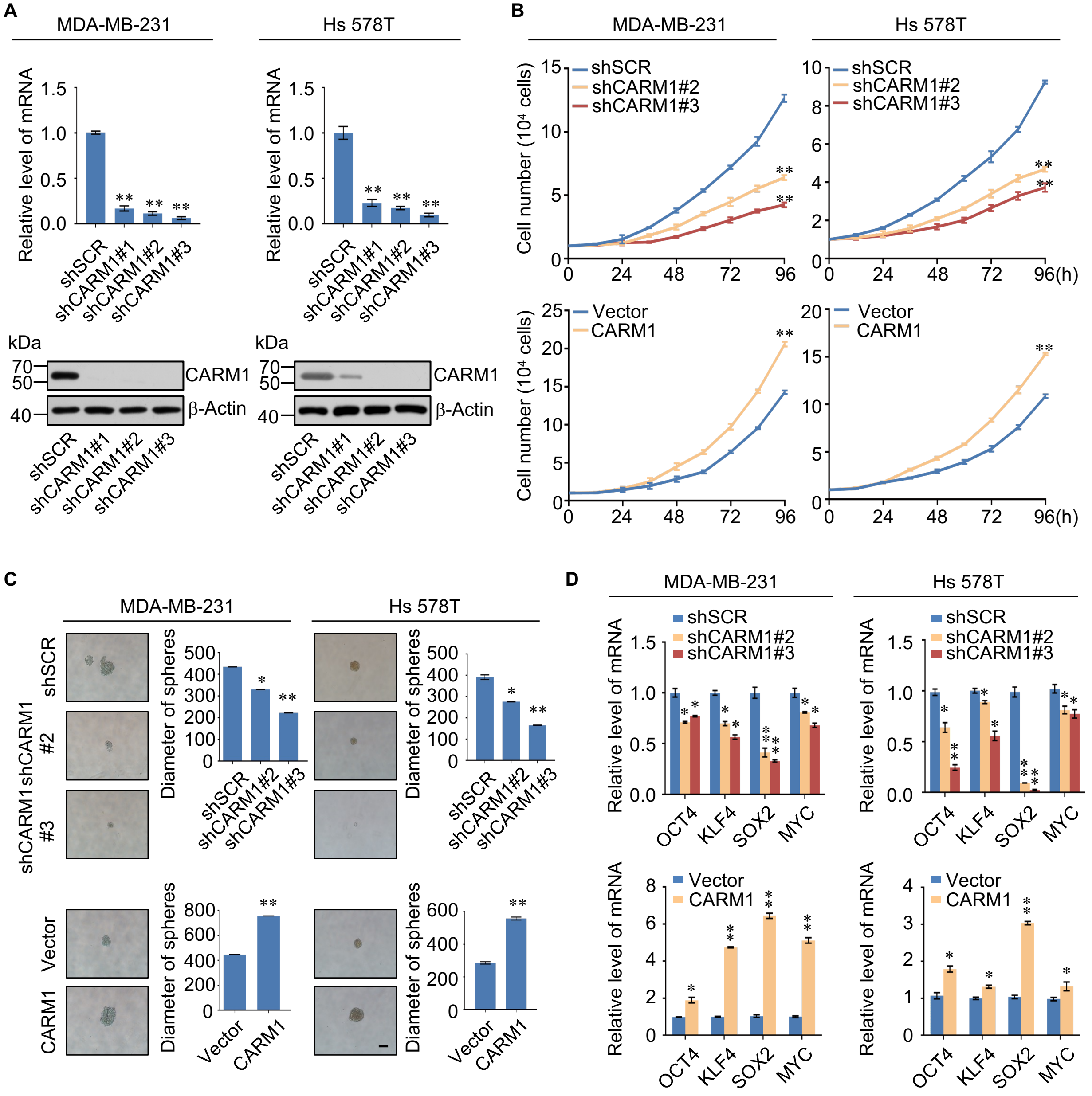
A
MDA-MB-231
Hs 578T
Relative level of mRNA
shSCR
shCARM1#1
shCARM1#2
shCARM1#3
kDa
CARM1
β-Actin
B
Cell number (10^4 cells)
shSCR
shCARM1#2
shCARM1#3
Vector
CARM1
96(h)
C
Diameter of spheres
shCARM1
D
OCT4
KLF4
SOX2
MYC

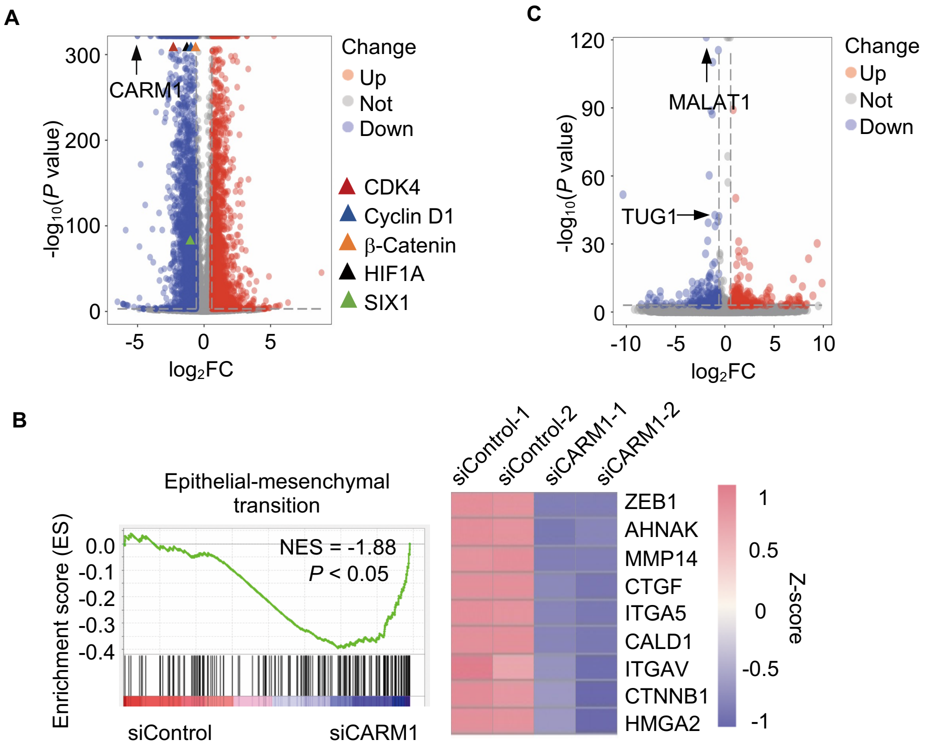
A
Change
Up
Not
Down
CDK4
Cyclin D1
β-Catenin
HIF1A
SIX1
CARM1
-log10(P value)
log2FC
300
200
100
0
-5
0
5
C
Change
Up
Not
Down
MALAT1
TUG1
-log10(P value)
log2FC
120
90
60
30
0
-10
-5
0
5
10
B
Epithelial-mesenchymal transition
NES = -1.88
P < 0.05
Enrichment score (ES)
0.0
-0.1
-0.2
-0.3
-0.4
siControl
siCARM1
siControl-1
siControl-2
siCARM1-1
siCARM1-2
ZEB1
AHNAK
MMP14
CTGF
ITGA5
CALD1
ITGAV
CTNNB1
HMGA2
1
0.5
0
-0.5
-1
Z-score

Supplementary Figure 4

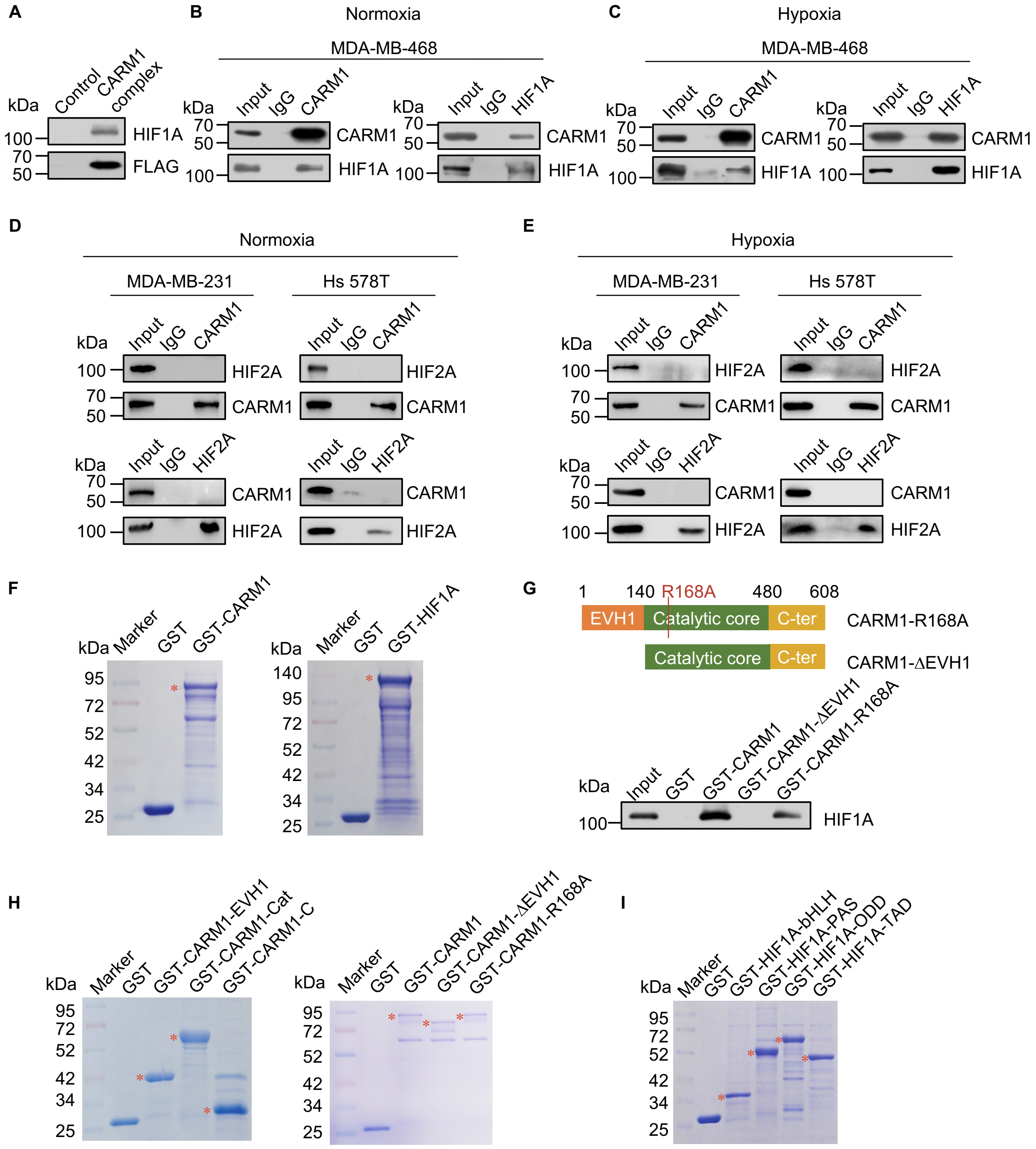

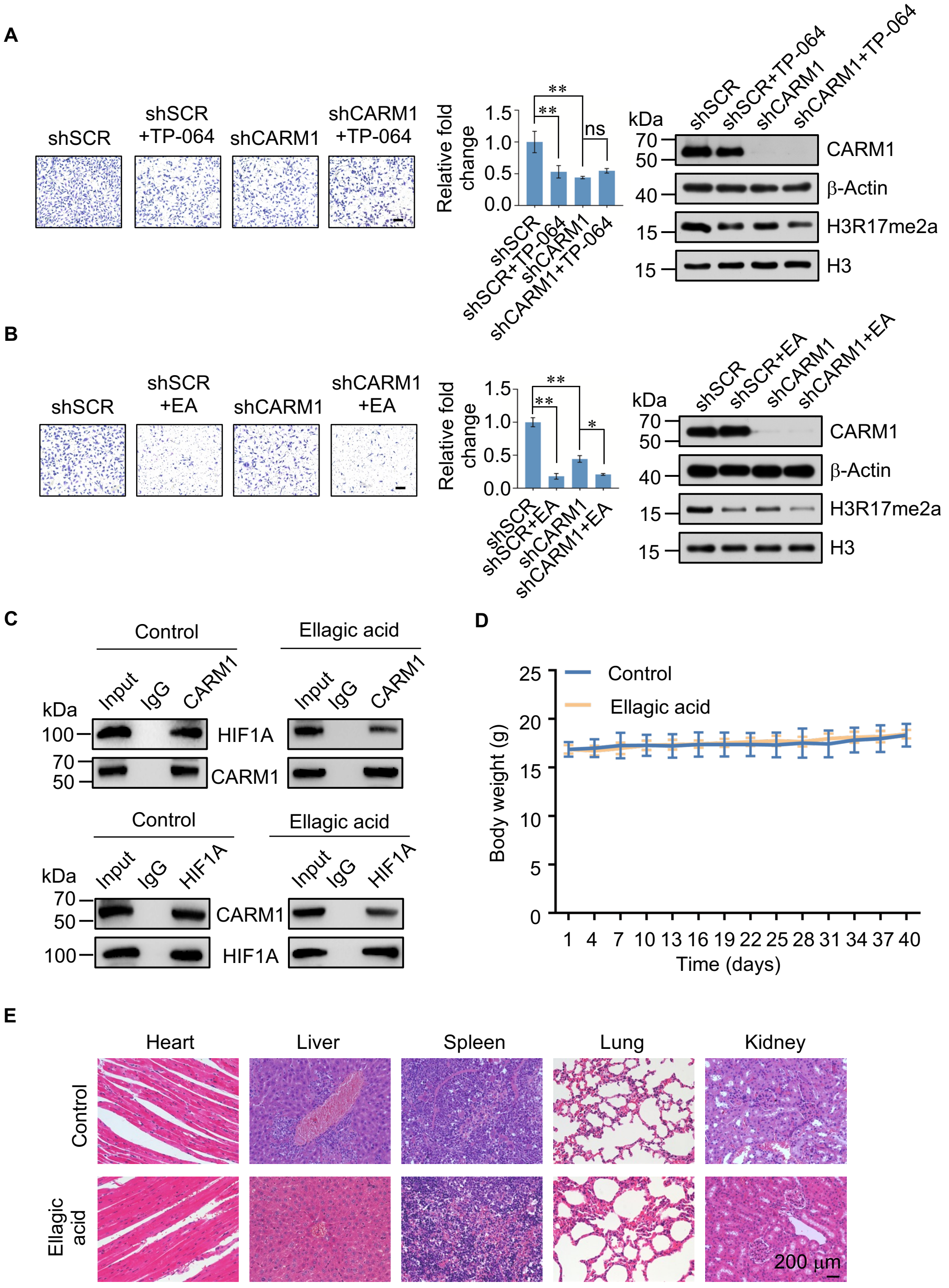
A
shSCR
shSCR +TP-064
shCARM1
shCARM1 +TP-064
Relative fold change
1.5
1.0
0.5
0.0
**
**
ns
shSCR
shSCR+TP-064
shCARM1
shCARM1+TP-064
kDa
70
50
40
15
15
CARM1
β-Actin
H3R17me2a
H3
B
shSCR
shSCR +EA
shCARM1
shCARM1 +EA
**
**
*
shSCR+EA
shCARM1+EA
C
Control
Ellagic acid
Input
IgG
CARM1
100
HIF1A
HIF1A
D
Body weight (g)
25
20
10
5
0
Control
Ellagic acid
1 4 7 10 13 16 19 22 25 28 31 34 37 40
Time (days)
E
Heart
Liver
Spleen
Lung
Kidney
Control
Ellagic acid
200 μm

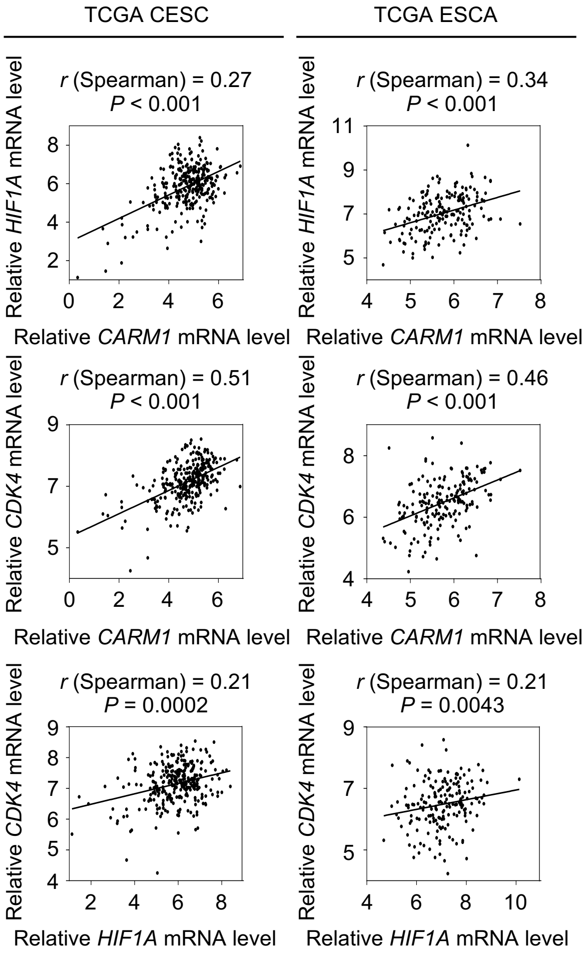
TCGA CESC
TCGA ESCA
r (Spearman) = 0.27
P < 0.001
r (Spearman) = 0.34
P < 0.001
Relative HIF1A mRNA level
Relative CARM1 mRNA level
r (Spearman) = 0.51
P < 0.001
r (Spearman) = 0.46
P < 0.001
Relative CDK4 mRNA level
Relative CARM1 mRNA level
r (Spearman) = 0.21
P = 0.0002
r (Spearman) = 0.21
P = 0.0043
Relative CDK4 mRNA level
Relative HIF1A mRNA level

Supplemental File 2

CARM1 drives triple-negative breast cancer progression by coordinating with HIF1A

Dandan Feng[1,2,‡], Jie Gao[3,‡], Ruiqiong Liu[4,‡], Wei Liu[2], Tianyang Gao[2], Yunkai Yang[1], Die Zhang[1], Tianshu Yang[5], Xin Yin[5], Hefen Yu[5], Wei Huang[5,*], and Yan Wang[1,2,5,*]

Contents:

Table S1. The shRNA target sequences used in the text.

Table S2. The RT-qPCR primers used in the text.

Table S3. The primers of ChIP assays used in the text.

Table S4. Proteomic analysis of FLAG-CARM1 in MDA-MB-231 cells.

Table S1. The shRNA target sequences used in the text.

**shRNA**	**Sequence**
shSCR	TTCTCCGAACGTGTCACGT *
shCARM1#1	GCAAGCAGTCCTTCATCATCA
shCARM1#2	CTATGACTTGAGCAGTGTTAT *
shCARM1#3	GCGGAAACATGTTTCCTACCA *
shHIF1A#1	CCGCTGGAGACACAATCATAT *
shHIF1A#2	GGGATTAACTCAGTTTGAACT
shHIF1A#3	GGAAATGAGAGAAATGCTTAC
shCDK4	ACAGTTCGTGAGGTGGCTTTA *

The shRNA sequences used in this study were listed as above and * indicates the shRNA chosen for further study.

Table S2. The RT-qPCR primers used in the text.

Gene	Strand	Sequence
PRMT1	F	CTTTGACTCCTACGCACACTT
PRMT1	R	GTGCCGGTTATGAAACATGGA
PRMT2	F	CCCAGAAGTGAATCGCAGGG
PRMT2	R	TGCAGTGGTTTGTCTCAGGATA
PRMT3	F	GTACCCTTCTCATACCCCAATGG
PRMT3	R	GACGAGCAGGTTCTGACATCT
CARM1	F	TCGCCACACCCAACGATTT
CARM1	R	GTACTGCACGGCAGAAGACT
PRMT5	F	CTGTCTTCCATCCGCGTTTCA
PRMT5	R	GCAGTAGGTCTGATCGTGTCTG
PRMT6	F	TACCGCCTGGGTATCCTTCG
PRMT6	R	CCTGTTCCGGCAACTCTACA
PRMT7	F	CCTGCTAAGCCCGTGCAAT
PRMT7	R	CAACGGGAGGGACGATGAC
PRMT8	F	CCTGCTAAGCCCGTGCAAT
PRMT8	R	TGGGCATAGGAGTCGAAGTAA

β-Actin	F	CATGTACGTTGCTATCCAGGC
β-Actin	R	CTCCTTAATGTCACGCACGAT
α-Catenin	F	GGGGATAAAATTGCGAAGGAGA
α-Catenin	R	GTTGCCTCGCTTCACAGAAGA
γ-Catenin	F	GGACAAGAACCCAGACTACC
γ-Catenin	R	GTGGCATCCATGTCATCTCC
Vimentin	F	GACGCCATCAACACCGAGTT
Vimentin	R	CTTTGTCGTTGGTTAGCTGGT
N-cadherin	F	TCAGGCGTCTGTAGAGGCTT
N-cadherin	R	ATGCACATCCTTCGATAAGACTG
OCT4	F	CTGGGTTGATCCTCGGACCT
OCT4	R	CCATCGGAGTTGCTCTCCA
KLF4	F	CCCACATGAAGCGACTTCCC
KLF4	R	CAGGTCCAGGAGATCGTTGAA
SOX2	F	GCCGAGTGGAAACTTTTGTCG
SOX2	R	GGCAGCGTGTACTTATCCTTCT
MYC	F	GGCTCCTGGCAAAAGGTCA

MYC	R	CTGCGTAGTTGTGCTGATGT
ATR	F	GGCCAAAGGCAGTTGTATTGA
ATR	R	GTGAGTACCCCAAAAATAGCAGG
CDK4	F	ATGGCTACCTCTCGATATGAGC
CDK4	R	CATTGGGGACTCTCACACTCT
Cyclin D1	F	GCTGCGAAGTGGAAACCATC
Cyclin D1	R	CCTCCTTCTGCACACATTTGAA
β-Catenin	F	AAAGCGGCTGTTAGTCACTGG
β-Catenin	R	CGAGTCATTGCATACTGTCCAT
ENO2	F	AGCCTCTACGGGCATCTATGA
ENO2	R	TTCTCAGTCCCATCCAACTCC
HIF1A	F	GAACGTCGAAAAGAAAAGTCTCG
HIF1A	R	CCTTATCAAGATGCGAACTCACA
HMOX1	F	AAGACTGCGTTCCTGCTCAAC
HMOX1	R	AAAGCCCTACAGCAACTGTCG
LAMC1	F	GGACTCCGCCCGAGGAATA
LAMC1	R	ACTTGAGACGCACATAGGTGA

MDM2	F	GAATCATCGGACTCAGGTACATC
MDM2	R	TCTGTCTCACTAATTGCTCTCCT
SIX1	F	CTGCCGTCGTTTGGCTTTAC
SIX1	R	GCTCTCGTTCTTGTGCAGGT
MALAT1	F	GGTTCAGAAGGTCTGAAGCTC
MALAT1	R	CCCAGAAGTGTTTACACTGCT
SNHG3	F	GGCTTTGGAATTTGGCATTT
SNHG3	R	GTTGCAGTGAGCAAGATCAT
SNHG12	F	CTGTGCTTTAAGATTCATGTTAC
SNHG12	R	CTGCTTCCCATAGAGATTGT
TUG1	F	AGGTAGAACCTCTATGCATTTTGTG
TUG1	R	ACTCTTGCTTCACTACTTCATCCAG
ZFAS1	F	AGCGGGTACAGAATGGATTT
ZFAS1	R	CAACAATAAACTCGTCAGGAGAT

Table S3. The primers of ChIP assays used in the text.

Gene	Strand	Sequence
ATR	F	TCTCAGAAAGAGGGACAAGA
ATR	R	ACAGTCCAGCGAGTAAAGG
CARM1	F	TAAGAAGGCAATGTGGGCAGAG
CARM1	R	CACCGACGATTTCCGATGAG
CDK4	F	CCCTCACAGCTTTCCACG
CDK4	R	CACCACCAGCATCCCATC
Cyclin D1	F	TGCTGAAGGCGGAGGAGA
Cyclin D1	R	CGTGGGTCTGGGCAACAAG
β-Catenin	F	GGAGGAAGGTCTGAGGAGC
β-Catenin	R	GGAAGGATAAGGAAAGGAGC
HIF1A	F	TGCCATTCATCCGTTCAG
HIF1A	R	AATCCAAGGTTGCCAAGT
LAMC1	F	CGGGGTAGGTGAGGGAAGCG
LAMC1	R	GGTCACGGCGAAGGCAAGG
MALAT1	F	TGCTCCGGTTCAGAAGGT
MALAT1	R	TGTTTACACTGCTCTGGGTCT

MAT2A	F	GAAAGCTATCCCGGCCAACG
MAT2A	R	ATGCAGACGCGAGGAGACG
MDM2	F	GGGAGTTCAGGGTAAAGG
MDM2	R	TTGACCAGCTCAAGAGGA
NEAT1	F	CAGAACAGCCACGCTCCA
NEAT1	R	CTATTCCTCCTGACTCCTCCAC
SIX1	F	TCATTGATTTGTGCGGAGTT
SIX1	R	GGAGTGGTGGGAGGAGAAG
VEGFA	F	GGTGCGTCCCCTTTCCC
VEGFA	R	CGGCTCATGTGACCCAGAC
Vimentin	F	TATTGCCGCCAAAGATTC
Vimentin	R	CTGGTGGAAGTCATTAAAGGTA
β-Actin	F	CTTGGCTGGGCGTGACTGT
β-Actin	R	CCCGTGATGAAGGCTACAAAC

Table S4. Proteomic analysis of FLAG-CARM1 in MDA-MB-231 cells.

Identified proteins	Band	Peptide number	Coverage	Peptides
DDB1	127kDa	37	20.18%	EMLGGEIIPR<br>IEVQDTSGGTTALR<br>IVVFQYSDGK<br>LEELHVIDVK<br>LVFSNVNLK<br>LYDGLFK<br>QSGESIDIITR<br>VTLGTQPTVLR<br>YLAIAPPIIK<br>SVLLLAYK<br>TVPLYESPR<br>IAVMELFR<br>GAVYSMVEFNGK<br>VVEELTR<br>LLASINSTVR<br>EATADDLIK<br>VIPLDR<br>YLLGDMEGR<br>TYEVSLR<br>LGDSQLVK<br>VYPEEAEPK<br>PTVIYSSNHKL<br>IETGLLSDR<br>EATADDLIK<br>LGDSQLVK<br>SSQVVVAVGR
HIF1A	110kDa	13	11.86%	ILALQNAQR

				NLLQGEELLR<br>SSADPALNQEVALK<br>TTVPEEELNPK<br>VESEDTSSLFDK<br>LFAEDTEAK<br>LTISYLR<br>LELVEK
OGT	103kDa	2	0.43%	TIIVTTR<br>IIFSPVAPK
STAT3	86kDa	2	1.935%	IVELFR<br>SAFVVER
MTA2	75kDa	2	2.695%	TLLADQGEIR<br>LPLATIVK
CARM1	63kDa	952	78.78%	GAAVDEYFR<br>IVVIPGK<br>LLTIGDANGEIQR<br>QPVVDTFDIR<br>SSNLLDLK<br>YTVNFLEAK<br>AILQNHTDFK<br>AILQNHTDFKDK<br>IEIPFK<br>MLESYLHAK<br>MLFNER<br>SIPTNTMHYGS<br>MMQDYVR
HDAC1	55kDa	6	11.41%	SIRPDNMSEYSK<br>YYAVNYPLR<br>YGEYFPGTGDLR<br>VMTVSFHK

				LFENLR<br>MEIYRPHK
NONO	54kDa	9	11.04%	AVVIVDDR<br>AAPGAEFAPNK<br>VELDNMPLR<br>AGEVFIHK<br>GIVEFSGK<br>TLAEIAK
RbAp48	48kDa	1	20.69%	GEFGGFGSVSGK
RbAp46	46kDa	4	8%	YMPQNPHIIATK<br>EMFEDTVEER<br>GEFGGFGSVTGK
CASP14	28kDa	10	16.53%	KTNPEIQSTLR<br>MAEAELVQEGK<br>TNPEIQSTLR<br>FQQAIDSR<br>MAEAELVQEGK<br>SNPRSLEEEK<br>TNPEIQSTLR
